# MCLRP: enhanced prediction of anticancer drug response through low-rank matrix completion and transcriptomic profiling

**DOI:** 10.1186/s12915-025-02457-8

**Published:** 2025-12-03

**Authors:** Kun Wang, Binhan Li, Miao Xu, Dailin Ding, Qihui Zheng, Geng Tian, Xueying Zeng, Jialiang Yang

**Affiliations:** 1 School of Mathematical Sciences, Ocean University of China, Qingdao, 266100 China; 2 Broad Institute of MIT and Harvard, 415 Main Street, Cambridge, MA 02142 USA; 3 Geneis Beijing Co., Ltd., Beijing, 100102 China; 4 Laboratory of Marine Mathematics, Ocean University of China, Qingdao, 266100 China; 5 Academician Workstation, Changsha Medical University, Changsha, 410219 China

**Keywords:** Low-rank matrix completion, Principal component analysis, Model interpretability, Drug-gene association networks, Gene co-mutations

## Abstract

**Background:**

Accurate prediction of anticancer drug responses remains a significant challenge due to the intricate interplay between genomic features and pharmacological mechanisms. We present Matrix Completion with Low-rank Regularization and Principal Component Analysis (MCLRP), a multimodal framework that synergistically integrates low-rank matrix completion with transcriptomic principal component analysis through dual-stream feature interaction. This innovative architecture not only leverages the similarities among drugs and mutation patterns in cell lines via matrix completion but also preserves gene-level interpretability of response patterns by incorporating gene expression data into the model.

**Results:**

Benchmarked against seven computational paradigms (including matrix completion, ridge regression, SRMF, and their hybrid variants) across the Genomics of Drug Sensitivity in Cancer (GDSC) and Cancer Cell Line Encyclopedia (CCLE) repositories, MCLRP demonstrated superior predictive performance for 75% of drug responses, alongside enhanced biological plausibility. Notably, the model identified imatinib as a potential therapeutic alternative for M14 melanoma cell lines through cross-drug response extrapolation, suggesting innovative strategies for overcoming doxorubicin resistance. Interestingly, our mutation-response mapping revealed that BRAF-mutated lineages exhibited a 4.7-fold increase in sensitivity (*p* < 1e-5) to AZ628 compared to wild-type lineages, with synergistic amplification (8.1-fold, *p* < 1e-7) observed in BRAF/PIK3CA co-mutants.

**Conclusions:**

These findings establish MCLRP as a dual-purpose predictive-analytical tool that not only enhances drug response forecasting but also uncovers mutation-specific pharmacological vulnerabilities through systems-level pattern recognition.

**Supplementary Information:**

The online version contains supplementary material available at 10.1186/s12915-025-02457-8.

## Key points

1. MCLRP synergizes low-rank matrix completion with transcriptomic principal component analysis via dual-stream feature interaction, capturing drug-drug and cell line-cell line gene expression similarities while preserving gene-level interpretability through transcriptomic integration.

2. MCLRP outperformed seven computational models (including matrix completion and hybrid methods) on GDSC and CCLE datasets, achieving superior predictions for 75% of drug responses and enhanced biological coherence.

3. MCLRP identified imatinib as a candidate therapy for M14 melanoma via cross-drug response extrapolation, offering a novel strategy to bypass doxorubicin resistance through systems-level pattern recognition.

4. MCLRP revealed BRAF mutations confer 4.7-fold increased sensitivity (*p* < 1e-5) to AZ628, amplified to 8.1-fold (*p* < 1e-7) in BRAF/PIK3CA co-mutants, demonstrating the model’s capacity to prioritize targetable genetic vulnerabilities.

5. MCLRP bridges predictive modeling with biological discovery, enabling both precision drug response forecasting and hypothesis-generating insights into mutation-driven therapeutic mechanisms.

**Supplementary Information:**

The online version contains supplementary material available at 10.1186/s12915-025-02457-8.

## Background

Patients with the same type of cancer often exhibit varied responses to the same medical treatment, highlighting the need for precision medicine in cancer. Precision medicine aims to identify the molecular cause of an individual’s cancer and tailor the treatment accordingly, thereby improving cancer treatment outcomes [1]. To achieve an optimized treatment, a critical step is to predict anticancer drug response based on individual’s molecular information [2]. However, predicting drug response at tissue level is challenging since the significant heterogeneity in cellular composition within cancer tissues. As a result, cancer cell lines have become a popular substitute for predicting drug response due to their homogeneous cellular composition.

In recent years, several large-scale high-throughput screening studies have cataloged the multi-omics and drug response data for hundreds of human cancer cell lines [3–5]. For example, initiated by the National Cancer Institute, the NCI-60 Human Tumor Cell Lines Screen was designed to screen up to 3000 small molecules on 60 different human tumor cell lines [6]. The Cancer Cell Line Encyclopedia (CCLE) screens the drug response profile of 24 anticancer drugs against 491 cancer cell lines together with the gene mutation and gene expression profiles of the cell lines [7, 8]. The Genomics of Drug Sensitivity in Cancer (GDSC) database stores the drug response information of 119 anticancer drugs on 655 cancer cell lines [9, 10]. These drug response databases have enabled in silico drug response prediction, drug target inference, and exploration of drug treatment mechanisms. These developments align with the growing use of bioinformatics and multi-omics approaches for anticancer drug discovery, which provide systematic strategies for integrating genomic and pharmacological data [11].

Various computational methods have been proposed to predict the response of anticancer drugs to cancer cell lines [12]. These methods can generally be divided into two categories: traditional (non-deep learning) methods and deep learning methods. Additionally, machine learning has been increasingly leveraged in virtual screening and molecular simulation to identify novel cancer therapeutics, highlighting its broad applicability beyond predictive modeling [13].

In the field of traditional non-deep learning methods, regression-based methods explicitly model drug responses against cancer cell lines as a regression function of representing features of cell lines, such as gene expression [4, 14, 15]. For instance, Sokolov et al. introduced the Generalized Elastic Net (GELnet), which incorporates gene pathway information into feature selection, directing the solutions towards sets of genes that are mechanistically interconnected [16]. Matrix factorization-based methods explicitly model the drug response matrix as the product of the cancer cell line feature matrix and the drug feature matrix [17]. For instance, Wang et al. proposed the similarity-regularized matrix factorization (SRMF) method, which incorporates the similarities of drugs and cell lines to predict drug response [18]. However, due to the non-convex nature of the optimization problem, the model employs the alternating minimization algorithm, which searches for local optima rather than the global optimum. Matrix completion-based methods explicitly model drug responses to cancer cell lines as matrix singular value decomposition problems [19, 20]. For example, Liu et al. proposed an ensemble method called Matrix Completion with Ridge Regression (MCRR) to predict anticancer drug responses in cancer cell lines [7]. MCRR combines a low-rank matrix completion model with a ridge regression model, leveraging the advantages of ensemble learning to enhance prediction performance. While matrix completion-based methods can successfully predict drug response, they do not provide a holistic view on the underlying connections between mutations, cell lines, and drug effects. Bayesian inference-based methods are statistical approaches commonly employed for drug response prediction, often utilizing a multi-task learning framework that utilizes Bayes’ Theorem to update model probabilities. For instance, Ammad-ud-din et al. extended the kernelized Bayesian matrix factorization method by incorporating component-wise multiple kernel learning (cwKBMF) to enable multi-task matrix factorization [21]. Previous studies have indicated that Bayesian inference-based methods exhibit lower prediction performance compared to regression-based methods [22].

Recently, multi-modal and multi-stage learning strategies have been explored for drug response prediction. For example, Song et al. proposed a multi-stage multi-modal framework integrating Simplified Molecular Input Line Entry System (SMILES), molecular graph representations, and omics features to enhance prediction accuracy [23]. Deep learning methods for drug response prediction can be divided into methods applied to neural network (NN) modules and other learning schemes unrelated to basic NN building blocks [24]. The schemes based on neural network modules include dense layers [25], convolutional layers [26–29], attention mechanism [30, 31], and graph neural network layers [32, 33]. PaccMann is a neural network based on multi-modal attention mechanism. Beyond single-drug response, deep neural networks have also been successfully applied to predict synergistic effects of drug combinations, demonstrating their flexibility and scalability in precision oncology [34]. Oskooei et al. [31] were the first to report its application in drug response prediction (DRP), exploiting late feature integration with attention mechanisms in both cell-line and drug sub-networks. On the cell-line path, gene expressions are encoded by self-attention to generate a gene attention (GA) vector. On the drug path, SMILES embeddings are combined with GA through contextual attention, where the GA vector serves as the context. DeepIC50 proposed by Joo et al. [27] is a multi-class classifier adopting an early integration strategy. By inputting cell line mutations and drug features (descriptors and fingerprints) into a three-layer convolutional neural network (CNN), followed by connecting a fully connected neural network (FC-NN) prediction module. Features are automatically extracted through the convolutional layer and classification prediction is made in the fully connected layer. When handling cell line drug response data, DeepIC50 can utilize the feature extraction ability of CNN to learn the associations between cell line and drug features, and then make multi-class classification predictions of drug responses. Methods unrelated to NN building blocks include autoencoders [35, 36], transfer learning [37, 38], and multi-task learning [39]. GeneVAE proposed by Dong et al. [35] is a method that uses variational autoencoder (VAE) to encode gene expression data. Its main function is to learn the low-dimensional representation of gene expression data. This model can obtain gene expression data from the CCLE dataset and compress the high-dimensional gene expression data into a low-dimensional latent representation through the architecture of VAE. In this process, VAE maps the input gene expression data to the latent space through the encoder, and then tries to reconstruct the original data from the latent representation through the decoder. By minimizing the reconstruction error and KL divergence, GeneVAE can learn an effective representation of the data. This low-dimensional representation can capture the key information in the gene expression data.

In terms of datasets, many models have mined drug-related information to improve the prediction results of the model. Jiang et al. [30] proposed a deep learning model DeepTTA based on the transformer architecture. By obtaining the SMILES information of drugs, the SMILES expression of drugs is divided into substructures of different granularities, and then these substructures are used for subsequent feature learning. Hostallero et al. [40] proposed two deep learning methods, BiG-DRP and BiG-DRP +, for drug response prediction. They utilize the chemical structure of drugs and the potential relationship between drugs and cell lines through bipartite graphs and heterogeneous graph convolutional networks. They use RDKit software to encode the obtained drug SMILES to generate drug descriptors, such as molecular weight, number of aromatic rings, etc. At the same time, descriptors with missing values are excluded. Eventually, 237 unique drugs are obtained, and each drug has a feature vector of length 198 to represent its drug descriptor. Most models obtain results based on cell lines. However, there are many biological differences between tumors and cell lines, and the effect on tumors cannot be obtained. Some methods begin to consider the cell line-tumor difference and try to combine tumor data in the training process or generalize the results obtained on cell lines to real tumors [40–42].

In terms of model interpretability, various deep learning (DL) methods have been proposed to endow the DL black box with interpretability. For instance, some methods [40, 41] identify the most relevant features (genes) by using feature-level attributes. On the other hand, some methods [43, 44] make the DL architecture interpretable by combining Gene Ontology (GO), signaling pathways or other prior information. However, beyond methodological elegance, interpretability is pivotal for precision medicine: it facilitates biomarker identification, supports hypothesis generation for therapeutic mechanisms, and builds clinician trust in computational predictions.

However, despite these advances, several limitations remain: (i) many approaches do not jointly capture drug–drug and cell line–cell line similarities within a unified framework; (ii) interpretability at the gene or pathway level is often limited, hindering translational utility; and (iii) mutation–drug association discovery is frequently performed as a separate downstream task rather than being directly informed by the predictive modeling process.

To address these gaps, we developed Matrix Completion with Low-rank Regularization and Principal Component Analysis (MCLRP), a multimodal framework that integrates low-rank matrix completion with transcriptomic principal component analysis (PCA). This design enables simultaneous modeling of drug–drug and cell–cell similarities while preserving gene-level interpretability through transcriptomic integration. By coupling predictive modeling with systematic mutation-response and pathway analyses, MCLRP not only improves predictive accuracy but also yields mechanistic insights that can guide drug repurposing and biomarker discovery in precision oncology.

In MCLRP, interpretability arises from linking PCA-derived latent biological axes to drug sensitivity patterns, and from validating these links through mutation-stratified analyses presented in the Results. For instance, we observe that BRAF-mutated cell lines show heightened sensitivity to specific kinase inhibitors, consistent with prior biological evidence.

## Results

We utilized our MCLRP model to analyze the CCLE and GDSC datasets. The integration of matrix completion and ridge regression (MCRR) demonstrated substantially higher prediction accuracy compared to other models, including cell line similarity network, drug similarity network, matrix completion (MC), and ridge regression (RR). In this study, we evaluated and compared the performance of MC, RR, MCRR, and SRMF based on metrics Pearson correlation coefficient (PCC) and Spearman correlation coefficient (SCC).

Beyond predictive performance evaluation, we systematically analyzed the predicted drug response matrix to gain biological insights. Specifically, we identified potential drug–cancer associations, examined mutation-driven sensitivity differences (including co-mutation effects), and performed pathway and tissue-level analyses to interpret the predicted response patterns in the context of precision oncology. These results are detailed in the following subsections.

### Prediction performance in the CCLE and GDSC datasets

**Fig. 1:**
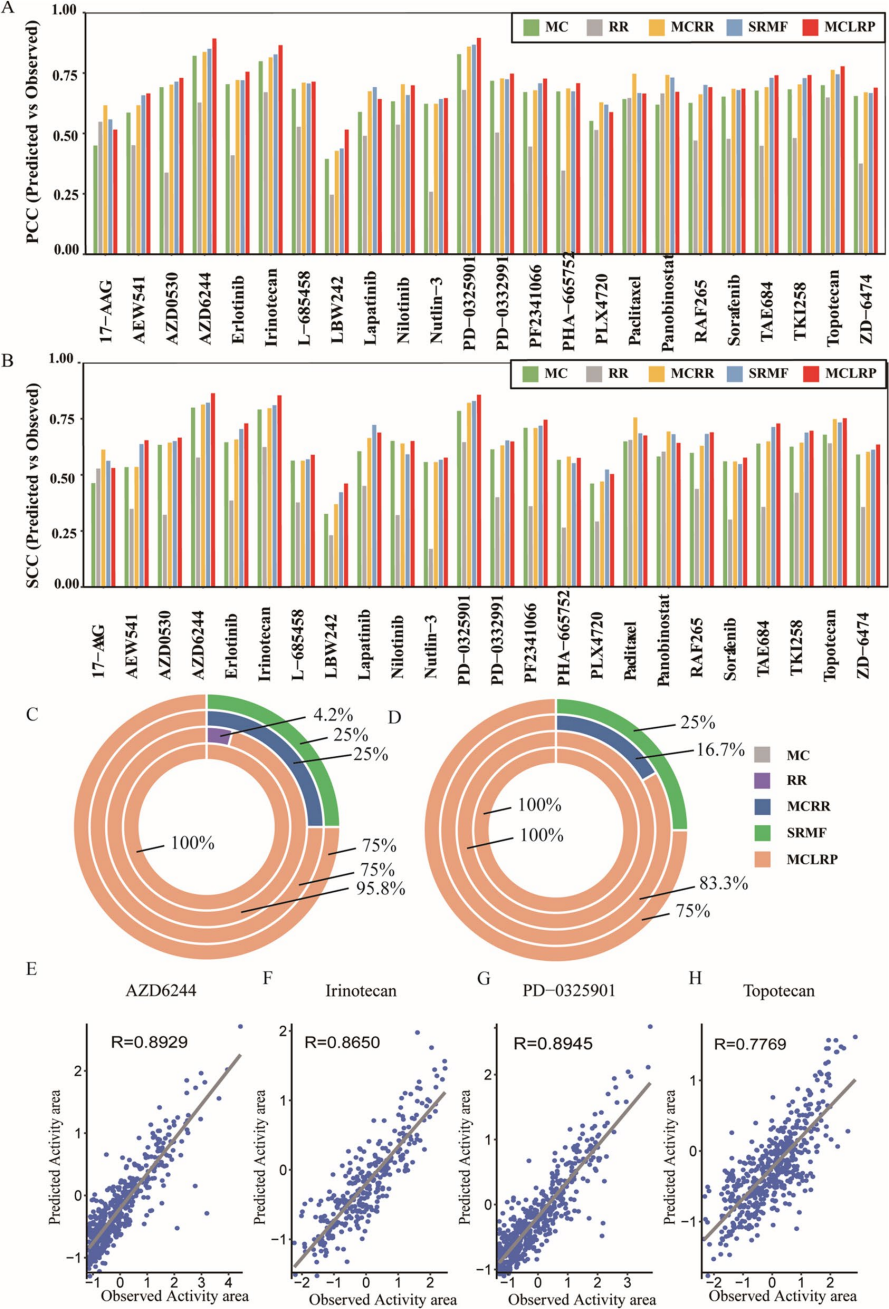
Assessments of prediction performance by the comparison of the five models in CCLE dataset (*n* = 491 cell lines, 24 drugs). PCC (**A**) and SCC (**B**) are calculated to measure the correlation between predicted and observed values. The MCLRP models outperform other models individually, as indicated by the highest proportion of drugs with optimal PCC (**C**) and SCC (**D**) values in the CCLE dataset. Scatterplots illustrating the observed and predicted activity areas for four drugs, namely, AZD6244 (**E**), Irinotecan (**F**), PD-0325901 (**G**), and Topotecan (**H**), using the MCLRP model

Figure 1A presents the comparison of the methods in CCLE in terms of PCC. Our model achieved PCC values higher than 0.6 in 21 drugs, higher than 0.7 in 12 drugs, and higher than 0.8 in 3 drugs, outperforming MCRR in 75% of the 24 drugs. Similarly, our model outperformed SRMF in 75% of the 24 drugs (see Fig. 1C). Notably, for the drug PD-0325901, our model achieved an impressive PCC of 0.895.

Figure 1B illustrates the comparison of the methods in the CCLE dataset based on SCC. Our model obtained SCC values higher than 0.6 in 17 drugs, higher than 0.7 in 7 drugs, and higher than 0.8 in 3 drugs, surpassing the performance of MCRR in 83.3% of the 24 drugs. Furthermore, our model outperformed SRMF in 75% of the drugs (see Fig. 1D). Particularly, for the drug AZD6244, the SCC reached 0.864. To further evaluate the performance of MCLRP, we created scatterplots of predicted and observed values for four drugs (see Fig. 1E–H). These scatterplots demonstrated the reasonability of the high PCC values, as the presence of outliers was minimal. Moreover, our model exhibited strong performance in terms of PCC and SCC for the remaining four datasets in GDSC (Additional file 1: Figs. S1, S2).

The observed improvement in PCC and SCC values compared to other models underscores the robustness and reliability of MCLRP. Higher PCC and SCC indicate stronger correlation and rank consistency between predicted and observed drug responses, which is critical for clinical translation. Accurate predictions ensure that the most effective drugs are prioritized for specific genetic contexts, reducing the risk of ineffective treatment and enhancing the potential for precision oncology applications.

### Comparison with deep learning-based methods

To further assess the competitiveness of MCLRP against state-of-the-art approaches, we compared it with two widely recognized deep learning-based models, DeepIC50 and GeneVAE, on the CCLE dataset. All models were trained using the drug–cell line response matrix as the primary input, while transcriptomic profiles were integrated as auxiliary features in accordance with each method’s design. Predictive performance was evaluated using Pearson correlation coefficient (PCC) and Spearman correlation coefficient (SCC) under the same 10 × tenfold cross-validation protocol.

**Fig. 2:**
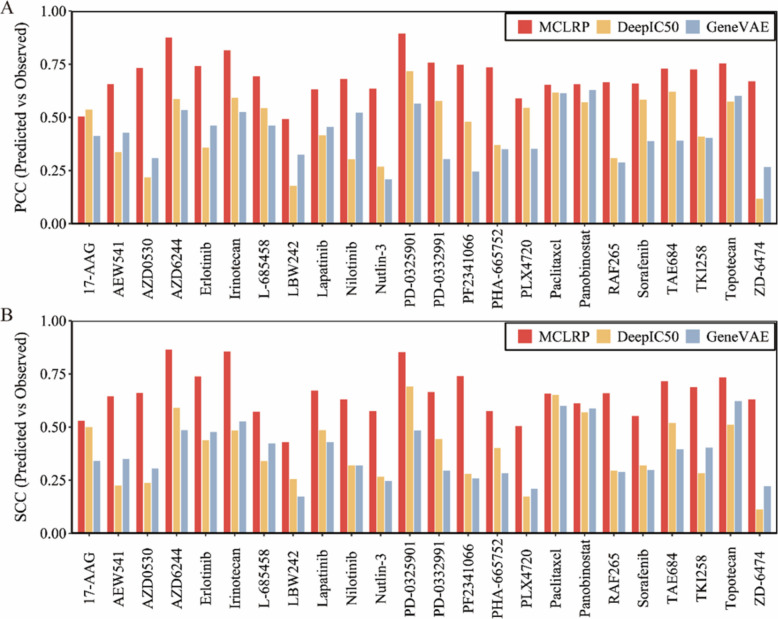
Comparison of MCLRP with two deep learning-based models (DeepIC50 and GeneVAE) on the CCLE dataset (*n* = 491 cell lines, 24 drugs). PCC (**A**) and SCC (**B**) are calculated to measure the correlation between predicted and observed values

Figure 2 summarizes the comparative results across 24 drugs. Overall, MCLRP consistently outperformed both DeepIC50 and GeneVAE in terms of both PCC and SCC. For example, MCLRP achieved PCC values ranging from 0.493 to 0.895, whereas DeepIC50 and GeneVAE showed substantially lower ranges of 0.117–0.718 and 0.208–0.628, respectively. A similar trend was observed for SCC, where MCLRP spanned 0.428–0.864, while DeepIC50 and GeneVAE only reached 0.112–0.690 and 0.172–0.622, respectively. When considering strong predictive performance (PCC > 0.70), MCLRP achieved this threshold in 12 out of 24 drugs, compared to 5 drugs for DeepIC50 and 4 drugs for GeneVAE. For SCC > 0.70, MCLRP reached this level in 7 drugs, while neither DeepIC50 nor GeneVAE achieved this performance in any drug. Furthermore, evaluation on the GDSC datasets confirmed the robustness of MCLRP, which consistently outperformed the deep learning-based baselines in both PCC and SCC metrics (Additional file 1: Fig. S3).

These findings highlight the robustness of MCLRP under limited-data conditions, where explicitly modeling the low-rank structure of the drug response matrix provides a clear advantage over purely nonlinear deep learning architectures.

Although deep learning approaches such as DeepIC50 and GeneVAE have demonstrated strong performance in large-scale and multimodal settings, their effectiveness diminishes in small and sparse datasets like CCLE. This is partly due to overfitting risks and the absence of additional drug-related features (e.g., chemical structure) commonly used in their original designs. In contrast, MCLRP leverages the intrinsic low-rank structure of the drug response matrix, reinforced by PCA-based feature compression to reduce noise, which enables robust prediction under data-limited conditions.

### Ablation studies

To further investigate the internal mechanisms driving the performance of MCLRP, we conducted ablation studies to quantify the contribution of its key components. Specifically, we compared the original model with two variants:

(i) MCLRP_NoPCA: the PCA-based dimensionality reduction for gene expression was removed, while the low-rank regularization and optimization framework were retained.

(ii) MCLRP_NoTrace: the low-rank regularization term was omitted, while PCA integration and the optimization framework were preserved.

All models were evaluated on the CCLE dataset using the same tenfold cross-validation repeated 10 times, and predictive performance was assessed with Pearson correlation coefficient (PCC) and Spearman correlation coefficient (SCC).

**Fig. 3:**
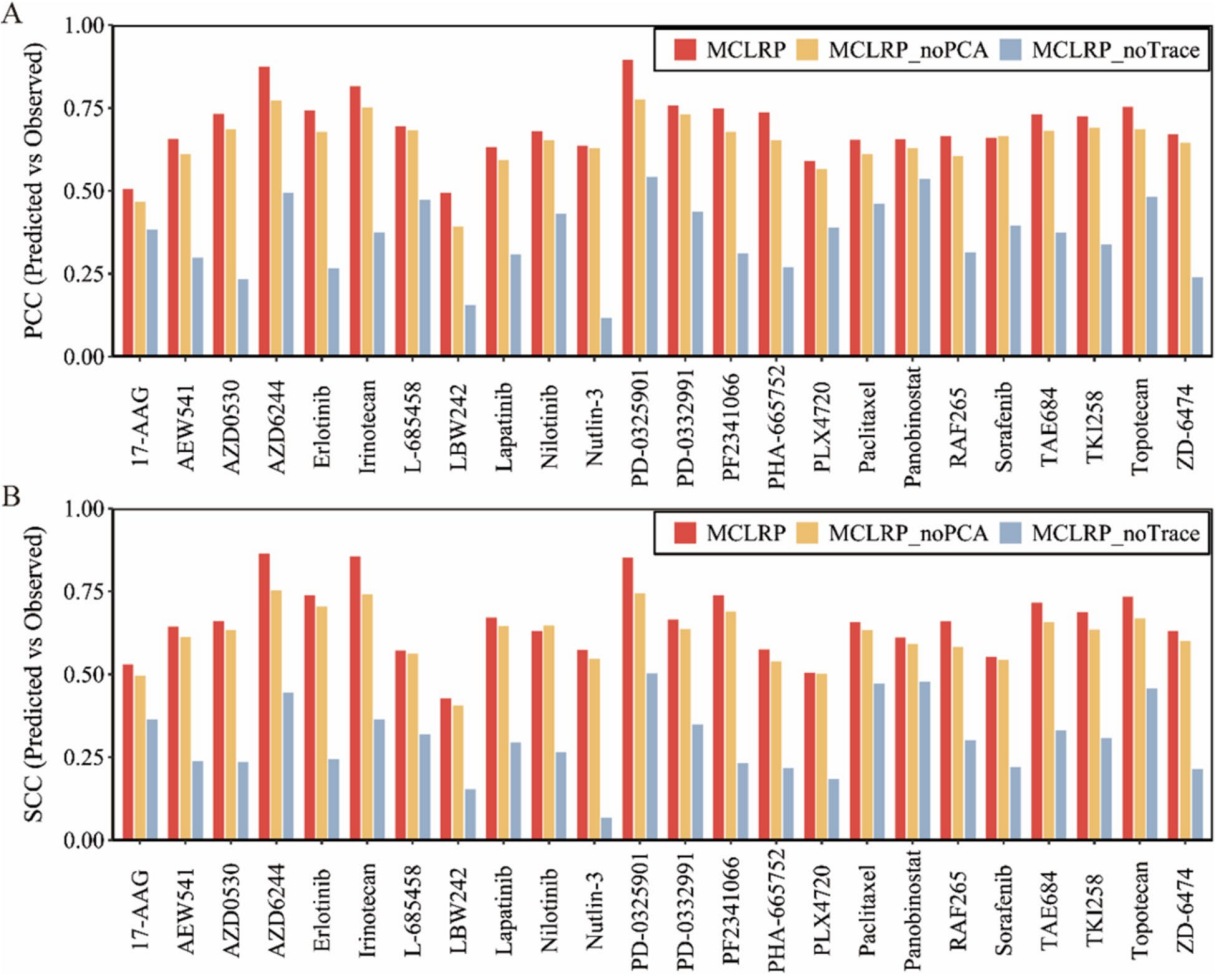
Ablation study evaluating the contribution of PCA integration and low-rank regularization in MCLRP on the CCLE dataset (*n* = 491 cell lines, 24 drugs). PCC (**A**) and SCC (**B**) values are calculated to measure the correlation between predicted and observed drug sensitivity across 24 drugs

Figure 3 shows the comparative performance of MCLRP and its two ablated variants across 24 drugs in the CCLE dataset. The original MCLRP consistently achieved the best results for both PCC and SCC. For example, PCC values of MCLRP ranged from 0.493 to 0.895, whereas MCLRP_NoPCA ranged from 0.391 to 0.775, and MCLRP_NoTrace dropped dramatically to as low as 0.116. Similar trends were observed for SCC: MCLRP spanned 0.428–0.864, while MCLRP_NoPCA ranged between 0.406 and 0.754, and MCLRP_NoTrace only reached 0.068–0.634. When considering strong predictive performance (PCC > 0.70), the full MCLRP achieved this level in 12 out of 24 drugs, compared to 7 drugs for MCLRP_NoPCA and none for MCLRP_NoTrace. For SCC > 0.70, MCLRP reached this threshold in 7 drugs, MCLRP_NoPCA in 3 drugs, and MCLRP_NoTrace in none. Moreover, similar trends were observed in the GDSC dataset, where MCLRP maintained superior performance compared to both ablated variants in terms of PCC and SCC (Additional file 1: Fig. S4).

These results demonstrate that both PCA-based transcriptomic integration and low-rank regularization are indispensable for the superior performance of MCLRP. Their synergy enables the model to effectively exploit molecular information and latent interaction structures, ultimately improving generalization and biological relevance.

### Predicting new drug-cancer associations in GDSC dataset

**Fig. 4:**
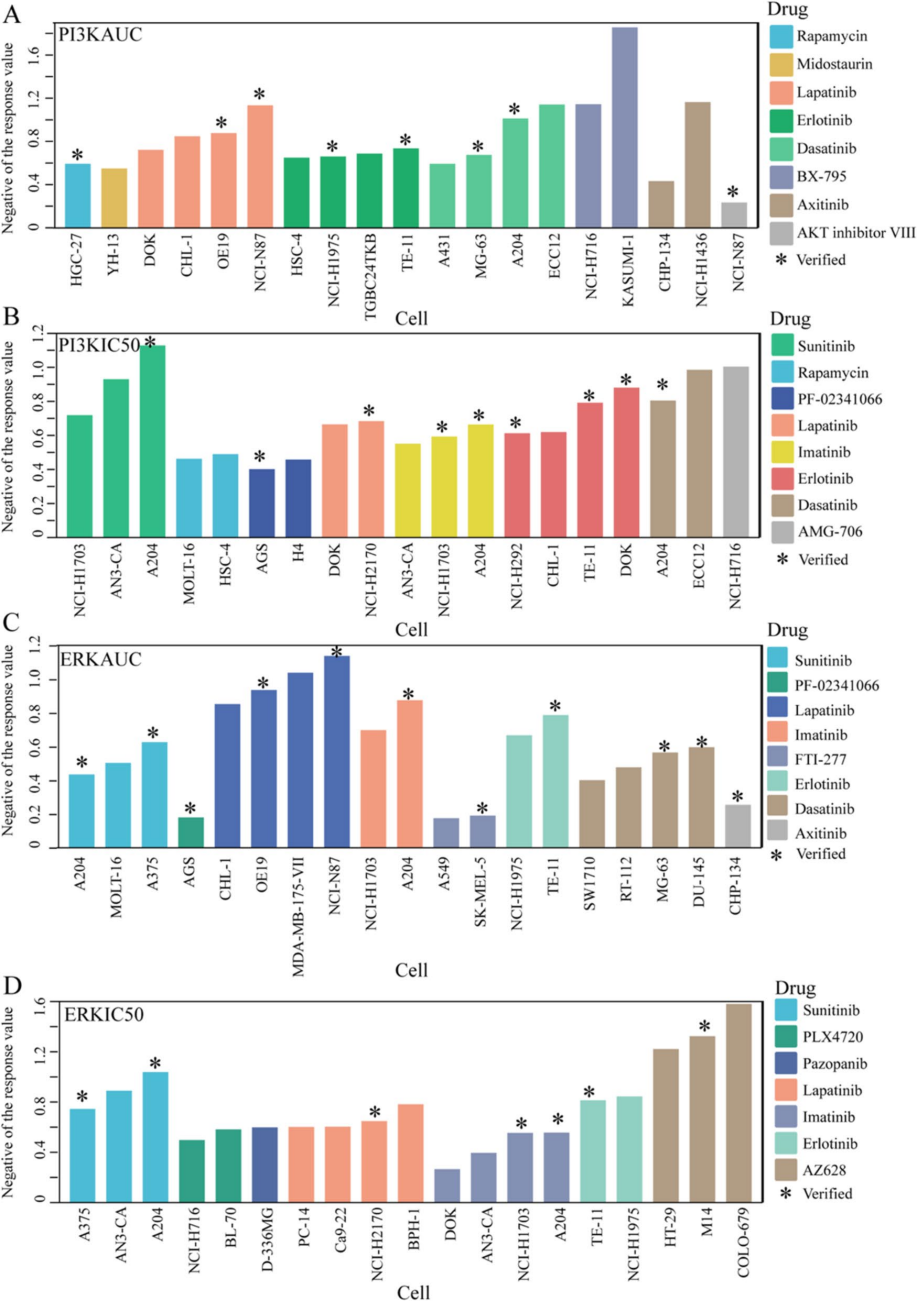
Drug sensitivity screening and evaluation by MCLRP in the GDSC datasets (sample size for each subpanel: **A** PI3KAUC: 655 cell lines, 28 drugs; **B** PI3KIC50: 655 cell lines, 29 drugs; **C** ERKAUC: 655 cell lines, 30 drugs; **D** ERKIC50: 655 cell lines, 32 drugs). The 30% threshold of observed values in the datasets **A** PI3KAUC, **B** PI3KIC50, **C** ERKAUC, and **D** ERKIC50 is used to screen the responsiveness of cancer cell lines to drugs in the predicted data. Only a subset of data is depicted in the figure

To further analyze the predictive performance of our model, we selected the predicted values corresponding to cell lines sensitive to each drug. We used the top 30% of observed values from the PI3KAUC, PI3KIC50, ERKAUC, and ERKIC50 datasets as thresholds and conducted case studies based on these selections (see Fig. 4A–D). The complete data for the predictive high sensitivity response values selected by different thresholds (30%, 35%, 40%) are in the Additional file 2: Tables S1–S4.

Numerous cancer cell lines with high drug sensitivity, as predicted by our model, have already been confirmed in previous studies. For instance, the loss of CSK or PTEN function in HER2-amplified gastric cancer cell lines, such as NCI-N87 and OE19, leads to lapatinib resistance in these cancer cells. However, this lapatinib resistance, induced by the loss of CSK or PTEN function, can be mitigated through the combined treatment of lapatinib with the PI3K inhibitor copanlisib and the MEK inhibitor trametinib [45]. Previous in vivo and in vitro studies explored the impact of erlotinib on the bone invasion of the human non-small cell lung cancer (NSCLC) cell line NCI-H292. The experimental outcomes indicated that erlotinib suppressed osteoclast activation by inhibiting tumor growth at the site of bone metastasis, reducing osteolytic factor production in tumor cells, attenuating osteoblast/stromal cell proliferation, and hindering osteoclast differentiation of mouse bone marrow cells. Consequently, this interference resulted in the inhibition of tumor-induced bone metastasis invasion [46]. Additionally, the effects of Dasatinib on A204 cells [47] and the effects of Lapatinib on OE19 cells [48] have also been experimentally validated.

### Drug-cancer association validation based on predicted response in GDSC dataset

**Fig. 5:**
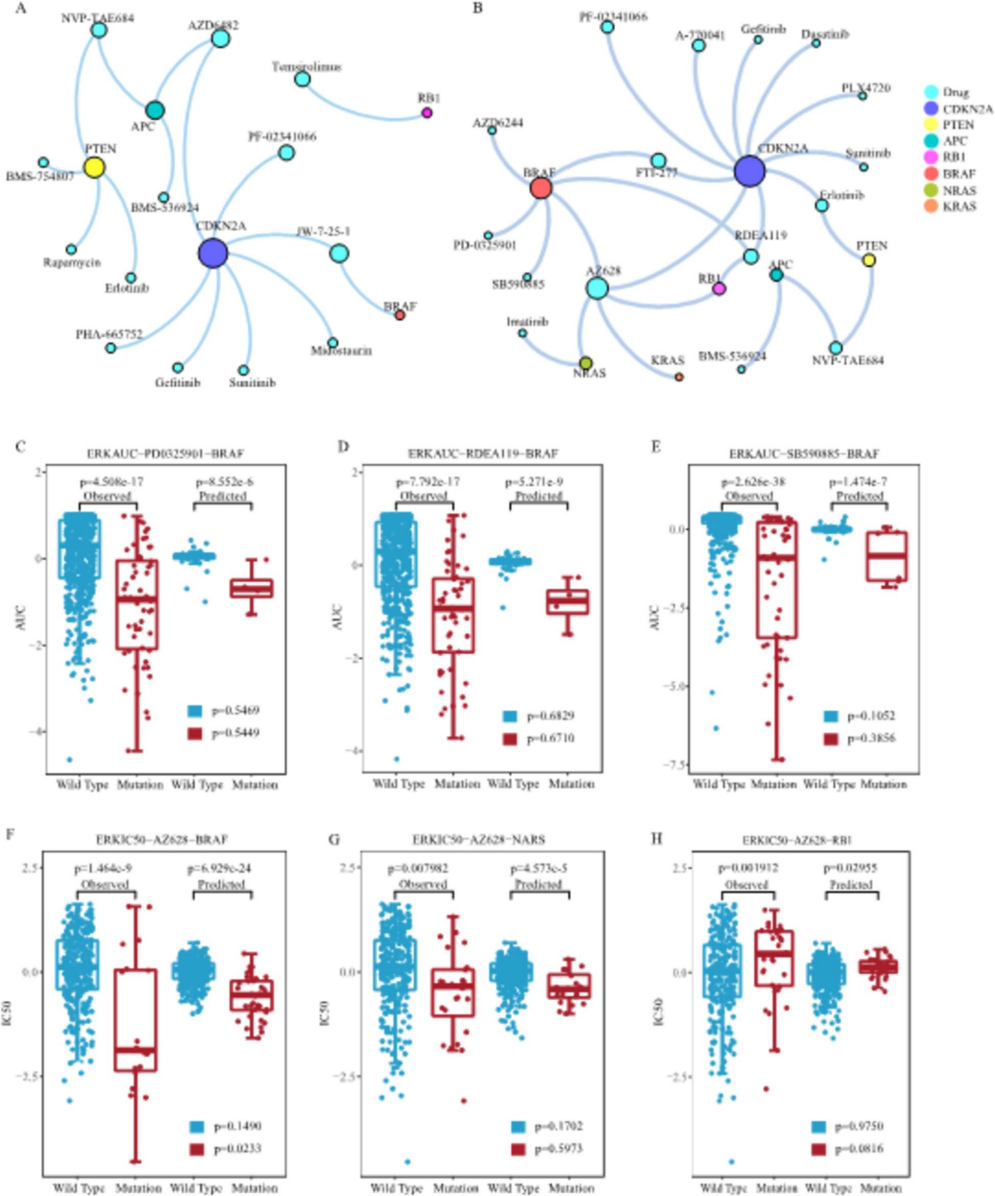
Drug–gene association networks and MCLRP effectiveness testing in the GDSC datasets (*n* = 655 cell lines, 119 drugs). Drug-gene association networks with mutant and wild-type *P* values less than 0.05 for both observed and predicted values in the PI3K pathway (**A**) and ERK pathway (**B**). Consistency identification results in ERKAUC between predicted and observed data for BRAF mutant and wild-type cell lines, specifically (**C**) PD-0325901, (**D**) RDEA119, and (**E**) SB590885. Consistency identification results in ERKIC50 between predicted and observed data for BRAF (**F**), NRAS (**G**), and RB1 (**H**) mutant and wild-type cell lines using AZ628

To further assess the model’s reliability in identifying missing values, we conducted an evaluation by classifying the cell lines as wild-type or mutant based on a specific gene. If the missing data displayed a similar or consistent distribution pattern to the existing data, the estimation of the missing values was deemed reliable. Using this criterion, our initial focus was on the predicted and observed responses of the BRAF gene to three MEK inhibitors: PD-0325901, RDEA119, and SB590885 in the ERKAUC dataset (see Fig. 5C–E).

BRAF, a serine/threonine kinase, plays a crucial role in regulating the mitogen-activated protein kinase (MAPK) cascade [49]. Under physiological conditions, this cascade regulates the expression of genes involved in various cellular functions, including proliferation [50]. Genetic alterations in the BRAF gene are prevalent in melanomas, thyroid cancers, histiocytic neoplasms, and a small portion of lung and colorectal cancers [51–53]. To investigate the relationship between gene mutations and responses to the three drugs, we analyzed the BRAF mutation profile of the cell lines. The predicted responses to the drugs were classified into two groups: BRAF mutation and BRAF wild-type. The same classification was applied to the observed responses. Figure 5C–E illustrates the predicted and observed responses, allowing us to assess the consistency between the predicted sensitivity of BRAF-mutant cell lines to the three drugs and the existing datasets. Notably, our predictions align with the response data from the cell lines and are consistent with previously published studies [54–56].

We further examined the predicted and observed response of BRAF, NRAS, and RB1 genes to the Raf inhibitor AZ628 in the ERKIC50 dataset (see Fig. 5F–H). AZ628 is known for inhibiting the activation of numerous tyrosine protein kinases. It has been demonstrated to induce cell cycle arrest and apoptosis in colon and melanoma cell lines harboring the B-RafV600E mutation [57]. By inhibiting RAF, AZ628 hinders the phosphorylation of MEK and ERK. In our study, we discovered that cell lines with BRAF mutations, NRAS mutations, and RB1 wild-type are responsive to AZ628. Importantly, the predicted data align with the observed data, demonstrating consistency in the trends between the two datasets.

Figure 5A, B illustrates the drug-gene association network, where both the wild-type and mutant *P* values are below 0.05 for both the observed and predicted values in the PI3K and ERK pathways. Within these pathways, the CDKN2A gene exhibits the most connections with drugs. CDKN2A is responsible for encoding the cell cycle regulatory protein p16^INK4a^, also known as the p16 gene, in the human genome. Mutations or deletions in the CDKN2A gene can result in the loss or reduction of p16^INK4a^ function, leading to aberrant activation of the cell cycle and promoting tumor initiation and progression [58]. These findings confirm the reliability of our results.

**Fig. 6:**
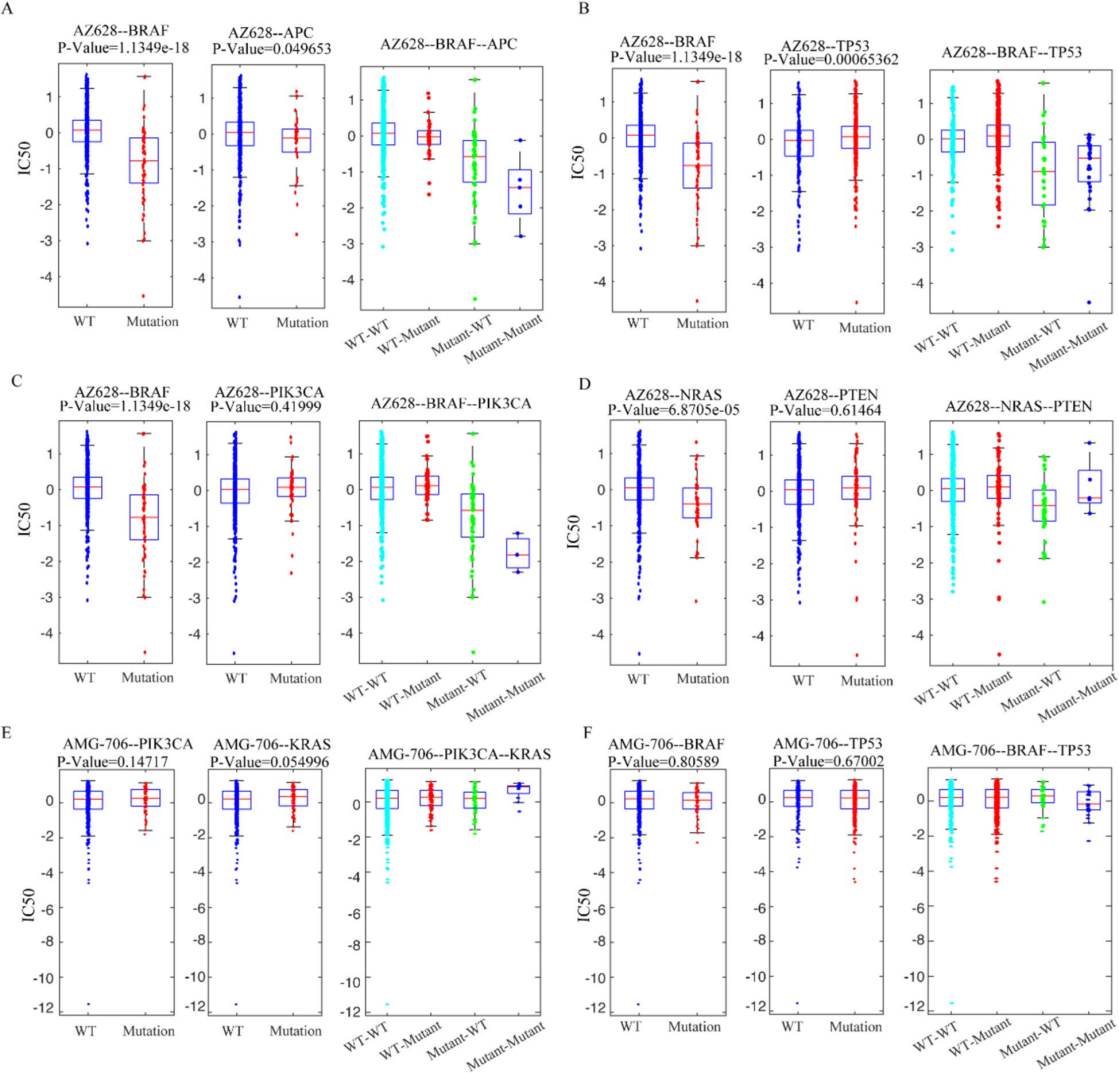
Response patterns of cell lines with no mutation, single gene mutation, and co-mutations on two specific genes for AZ628 or AMG-706(*n* = 655 cell lines).** A** Response of BRAF and APC mutant and wild-type cell lines to AZ628. **B** Response of BRAF and TP53 mutant and wild-type cell lines to AZ628. **C** Response of BRAF and PIK3CA mutant and wild-type cell lines to AZ628. **D** Response of NRAS and PTEN mutant and wild-type cell lines to AZ628. **E** Response of PIK3CA and KRAS mutant and wild-type cell lines to AMG-706. **F** Response of BRAF and TP53 mutant and wild-type cell lines to AMG-706

To further investigate the drug sensitivity of cancer cell lines, we utilized two genes to classify the cell lines into four categories: both genes being wild-type, one gene being wild-type and the other gene being mutated, both genes being mutated. Subsequently, we observed that the drug responses varied among cell lines with different gene combinations. In the ERKIC50 dataset, the drug AZ628 exhibited significant differences in cell lines containing single gene mutations of BRAF or APC, with a higher sensitivity observed in cell lines harboring mutations in both BRAF and APC genes (refer to Fig. 6A). Similarly, AZ628 demonstrated significant differences in cell lines with single gene mutations of BRAF or TP53, where cell lines with BRAF mutations and TP53 wild-type exhibited increased sensitivity to AZ628 (see Fig. 6B). Furthermore, AZ628 displayed significant differences in cell lines with BRAF mutations compared to wild-type counterparts, while no significant differences were observed in cell lines with PIK3CA gene mutations compared to wild types. Notably, cell lines with mutations in both BRAF and PIK3CA genes showed enhanced sensitivity to AZ628 (see Fig. 6C). In the case of cell lines containing mutant and wild-type forms of the NRAS gene, AZ628 exhibited significant differences, while no significant differences were observed in cell lines with mutant and wild-type forms of the PTEN gene. Cell lines with NRAS gene mutations and wild-type PTEN gene exhibited increased sensitivity to AZ628 (see Fig. 6D). On the other hand, AMG-706 did not show significant differences in cell lines containing single gene mutations of PIK3CA or KRAS. However, cell lines with both KRAS and PIK3CA gene mutations displayed increased resistance to AMG-706 (see Fig. 6E). Similarly, AMG-706 did not exhibit significant differences in cell lines with single gene mutations of BRAF or TP53, but cell lines with both BRAF and TP53 gene mutations were more sensitive to AMG-706 (see Fig. 6F).

Furthermore, Additional file 2: Table S5 presents the results displaying the relationship between additional drugs and co-mutations on one or more genes.

These co-mutation findings may have potential therapeutic relevance. For example, the observed increased sensitivity of BRAF/PIK3CA double-mutant cell lines to AZ628 raises the hypothesis that combined inhibition of RAF and PI3K pathways could be explored in cancers harboring these alterations. Co-occurrence of MAPK and PI3K pathway changes has been reported in multiple cancer types, which has been suggested as a factor associated with aggressive behavior and resistance to single-agent therapy. Our results provide computational evidence consistent with this notion, suggesting that these co-mutations could represent a context worth further experimental investigation rather than definitive clinical recommendation.

## Discussion

Based on the MCLRP model, we analyzed the top 1000 genes (Additional file 2: Tables S6–S9) corresponding to each drug in the five datasets in order to use the gene expression profile. It is worth noting that many of these selected genes are well-established biomarkers of drug sensitivity. For instance, Erlotinib inhibits tyrosine kinase activity, preventing HER1/EGFR phosphorylation and downstream signaling events, thereby blocking tumorigenesis driven by aberrant HER1/EGFR signaling [59]. Lapatinib effectively suppresses ERBB2 tyrosine phosphorylation, leading to growth arrest or apoptosis in ERBB2-dependent tumor cell lines [60]. In the CCLE dataset, KIT emerges as one of the top genes associated with 12 drugs, indicating its potential as a therapeutic target for various anticancer drugs, including nilotinib [61] and sorafenib [62]. The consistency with the previous findings and drug applications promised our MCLRP method and results to be used as an important resource for the drug discovery.

The rationale for introducing PCA-reduced gene expression profiles into the matrix completion framework is to overcome the limitations of relying solely on the low-rank structure of the response matrix. Pure matrix completion captures global interaction patterns but ignores rich biological information, which may reduce accuracy when data are sparse or noisy. By incorporating PCA components, which retain the most informative variation from high-dimensional gene expression data, MCLRP leverages biologically meaningful signals while reducing redundancy and overfitting. This integration aligns the latent structure of drug responses with interpretable molecular variation, thereby improving both robustness and predictive power compared to matrix completion alone.

**Fig. 7:**
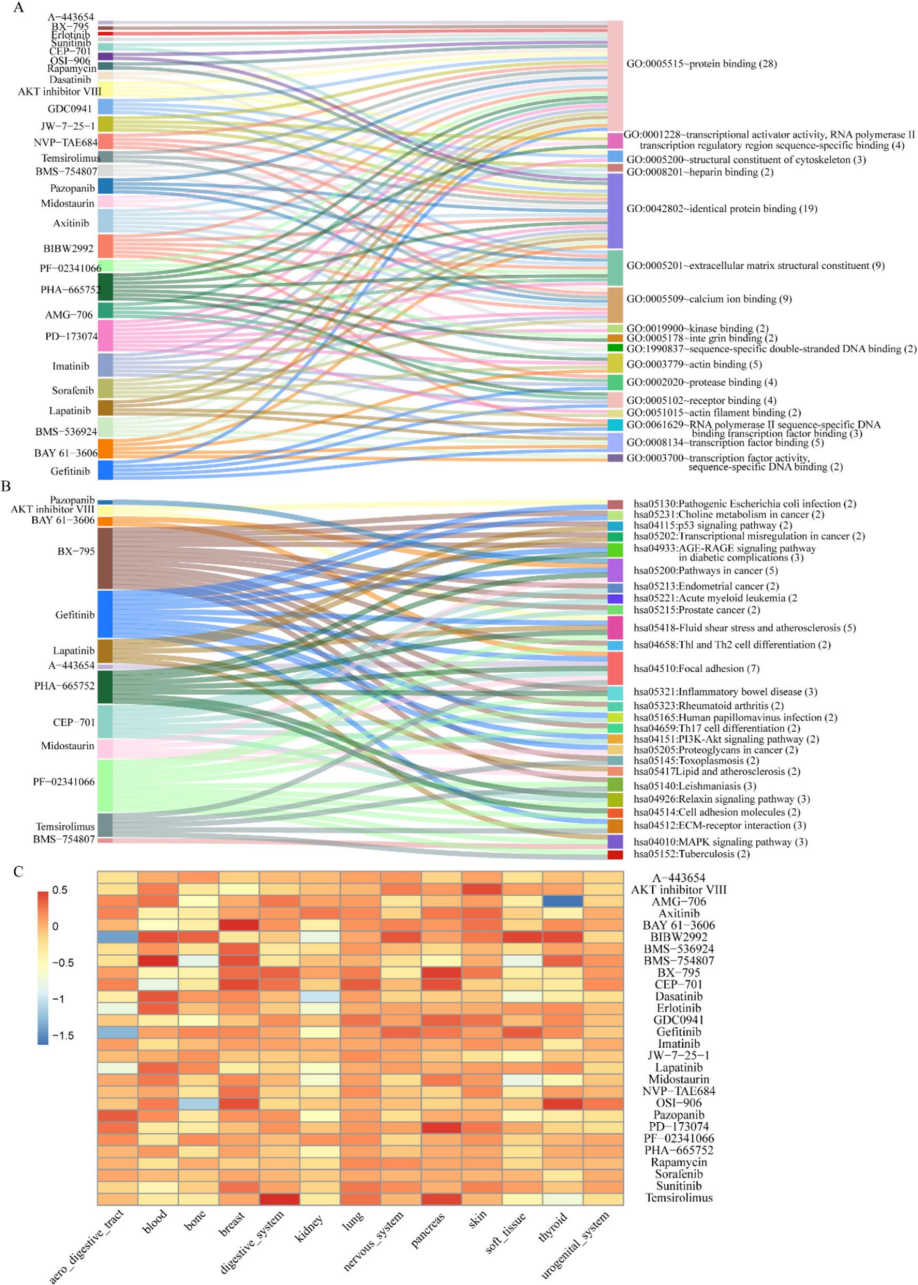
Functional enrichment and mean response patterns in the curated PI3KAUC dataset (*n* = 655 cell lines, 28 drugs).** A**, **B** Sankey plots illustrating enriched GO-MF (molecular function) and KEGG pathways derived from the top 1000 genes per drug, showing terms associated with at least two drugs in the PI3KAUC dataset. **C** Mean drug response values across tissues, corresponding drugs, and cell lines in the PI3KAUC dataset

We also conducted functional enrichment analysis of the top 1000 genes for each drug using DAVID (https://david.ncifcrf.gov/home.jsp) and presented the terms with a Benjamin false discovery rate of < 0.05 in Additional file 2: Tables S10-S13. In Fig. 7A, B, we depicted the Gene Ontology (GO) terms belonging to the molecular function (MF) category and the Kyoto Encyclopedia of Genes and Genomes (KEGG) terms that were associated with at least 2 drugs in the PI3KAUC dataset. The results showed that many of these functions have close associations with cancer which supports the efficiency of the MCLRP model. For instance, identical protein binding (GO:0042802) refers to the formation of a complex through interaction between two or more identical protein molecules. In numerous tumors, overexpression or mutation of proteins can lead to aberrant identical protein binding, which promotes the proliferation and survival of tumor cells. An example is HER2/neu, a highly expressed protein in breast cancer, which promotes tumor cell growth and survival by forming dimers or multimers [63].

To further assess the predictive capability of our model, we classified the cell lines based on their corresponding tissues and analyzed the tissue-specific drug effects on these tissues. We calculated the average response value of each drug across all cell lines within a particular tissue as the representative response value of that drug for the tissue (see Fig. 7C). For instance, epidermal growth factor receptor (EGFR) or mitogen-activated protein kinase (MEK1/2) kinase activity hinders the growth of renal cell carcinoma cells and enhances the growth inhibitory effects of the mammalian target of rapamycin (mTOR) inhibitor rapamycin. Furthermore, the combination of mTOR and other upstream inhibitors has demonstrated significant potential for the treatment of renal cell carcinoma [64]. The illustration of tissue specific drug effect is critical for the precision medicine development during cancer treatment.

In addition, we also have carried out thorough evaluations on the GDSC V2 version. Specifically, we focused on 256 drugs and employed the ten-times ten-fold cross-validation approach for assessment on both the area under the curve (AUC) and half maximal inhibitory concentration (IC50) datasets within GDSC V2. On the GDSC V2 AUC dataset, the results were as follows: with respect to the PCC, 55 out of 256 drugs had a PCC greater than 0.8, and 106 out of 256 drugs had a PCC greater than 0.7. Regarding the SCC, 36 out of 256 drugs had an SCC greater than 0.8, and 90 out of 256 drugs had an SCC greater than 0.7. On the GDSC V2 IC50 dataset, in terms of the PCC, 118 out of 256 drugs had a PCC greater than 0.8, and 237 out of 256 drugs had a PCC greater than 0.7. Concerning the SCC, 99 out of 256 drugs had an SCC greater than 0.8, and 234 out of 256 drugs had an SCC greater than 0.7. When compared to the performance on older databases, our model clearly demonstrates enhanced performance on the GDSC V2 dataset. The improvement is evident across multiple metrics and datasets, signifying that our model is better able to handle the complexity and diversity of the data in GDSC V2. This not only validates the effectiveness of our model’s design and training but also highlights its potential for more accurate and reliable predictions in the context of cancer drug response studies.

Although the current implementation predicts responses for observed drug–cell line pairs, MCLRP could be adapted to handle unseen drugs or cell lines by integrating similarity-based transfer learning or embedding representations. For example, incorporating chemical structure descriptors for drugs and pathway-level features for cell lines would allow the model to estimate responses for new entities using learned relationships from related samples.

We also point out the limitations of the proposed MCLRP model. Firstly, the experimental data evaluation criteria for cell lines and drugs were inconsistent, and the degrees of data missing varied, which affected the model’s universality across different datasets and the comparison results. During the processing of gene expression data, we only selected genes with known data in cancer cell lines and deleted genes with missing data. This might lead to information loss and impact the model’s learning and grasping of certain gene-related features. Secondly, MCLRP model constructed based on the matrix completion algorithm may have some inherent limitations. It can only interpolate drug responses and is unable to predict new cell lines and new drugs, which, to some extent, restricts the application scope of the model. Moreover, it may not be able to fully and accurately capture and model complex relationships between cell lines and drugs.

To address these limitations, we can consider the following possible improvement directions. First, we expand and optimize the model. For example, we can introduce more biological feature information, such as tumor microenvironment-related information and dynamic changes in gene expression, to enhance the model’s adaptability to unknown situations. Second, we draw on other advanced methods. For example, we can combine our model with some methods capable of handling new cell lines and new drugs, and realize the prediction of the responses of unseen cell lines or patient tumors by integrating the advantages of different methods.

Interpretability remains a key challenge in predictive modeling. While MCLRP preserves some biological interpretability through PCA, more granular attribution of gene-level contributions would increase its utility for mechanistic insights. Future extensions could leverage explainable AI (XAI) techniques, such as feature attribution, attention-based visualization, or Shapley value analysis, to provide transparent decision-making and enhance trust in predictive models for precision medicine.

## Conclusions

In this study, we proposed an innovative model by integrating matrix completion with low-rank regularization and principal component analysis using gene expression profiles to predict drug response. Through both genome-wide analysis and case studies, we addressed multiple objects, such as the identification of novel drug-cancer associations, the exploration of the tissue specific and mutation sensitive drug responses, and the investigation of the relationship between pathways and drugs. We anticipated that MCLRP method holds great potential significantly contribute to the discovery of precision medicine for cancer treatment across many aspects.

## Methods

### Data collection and preprocessing

We selected CCLE and GDSC because they are widely recognized benchmark datasets for drug response prediction, covering diverse drugs, pathways (e.g., PI3K and ERK), and experimental settings. Their complementary characteristics allow for evaluating the generalizability and robustness of MCLRP across heterogeneous data sources. The CCLE and GDSC projects represent two prominent publicly available resources for studying the anticancer drug response across hundreds of cancer cell lines. These resources provide comprehensive genomic and transcriptomic information, including data on mutations, gene expression, and copy number variations. The CCLE dataset can be accessed at https://www.broadinstitute.org/ccle, while the GDSC dataset is available at https://www.cancerrxgene.org. The CCLE dataset comprises data from 491 cancer cell lines, encompassing drug response profiles for 24 anticancer drugs, which could be represented as a matrix of size 491 × 24, resulting in a total of 11,784 entries. To handle missing data in the drug response matrix, we initially replaced missing entries with zero for matrix initialization; however, these entries were later estimated through the matrix completion process, ensuring that imputation was performed in a model-based manner rather than remaining as zeros. For gene expression profiles, we retained only genes with complete measurements across cell lines to prevent bias introduced by missing values. Each entry denotes the drug response measured by the activity area, where higher values indicate greater sensitivity of the cell line to the drug. Throughout the dataset, a total of 424 entries (3.6% of the matrix) are missing. Additionally, the CCLE dataset includes mutation and expression profiles of 18,900 genes. On the other hand, the GDSC dataset consists of data from 655 cancer cell lines, featuring drug response profiles for 119 anticancer drugs, including those targeting the PI3K and ERK pathways. Each entry denotes the drug response measured by the AUC or IC50, where lower values indicate greater sensitivity of the cell line to the drug. In our study, we specifically utilized the expression profiles of 12,072 genes that were complete without any missing data. A comprehensive overview of the dataset information is provided in Table 1.

**Table 1:** Detailed information related to datasets

Dataset	Pathway	Evaluation index	Drugs	Cells	Missing rate	Genes
CCLE	N/A	Activity area	24	491	3.6%	18,900
GDSC	PI3K	AUC	28	655	27.31%	12,072
GDSC	PI3K	IC50	29	655	26.68%	12,072
GDSC	ERK	AUC	30	655	26.02%	12,072
GDSC	ERK	IC50	32	655	26.32%	12,072

### Problem definition

**Fig. 8:**
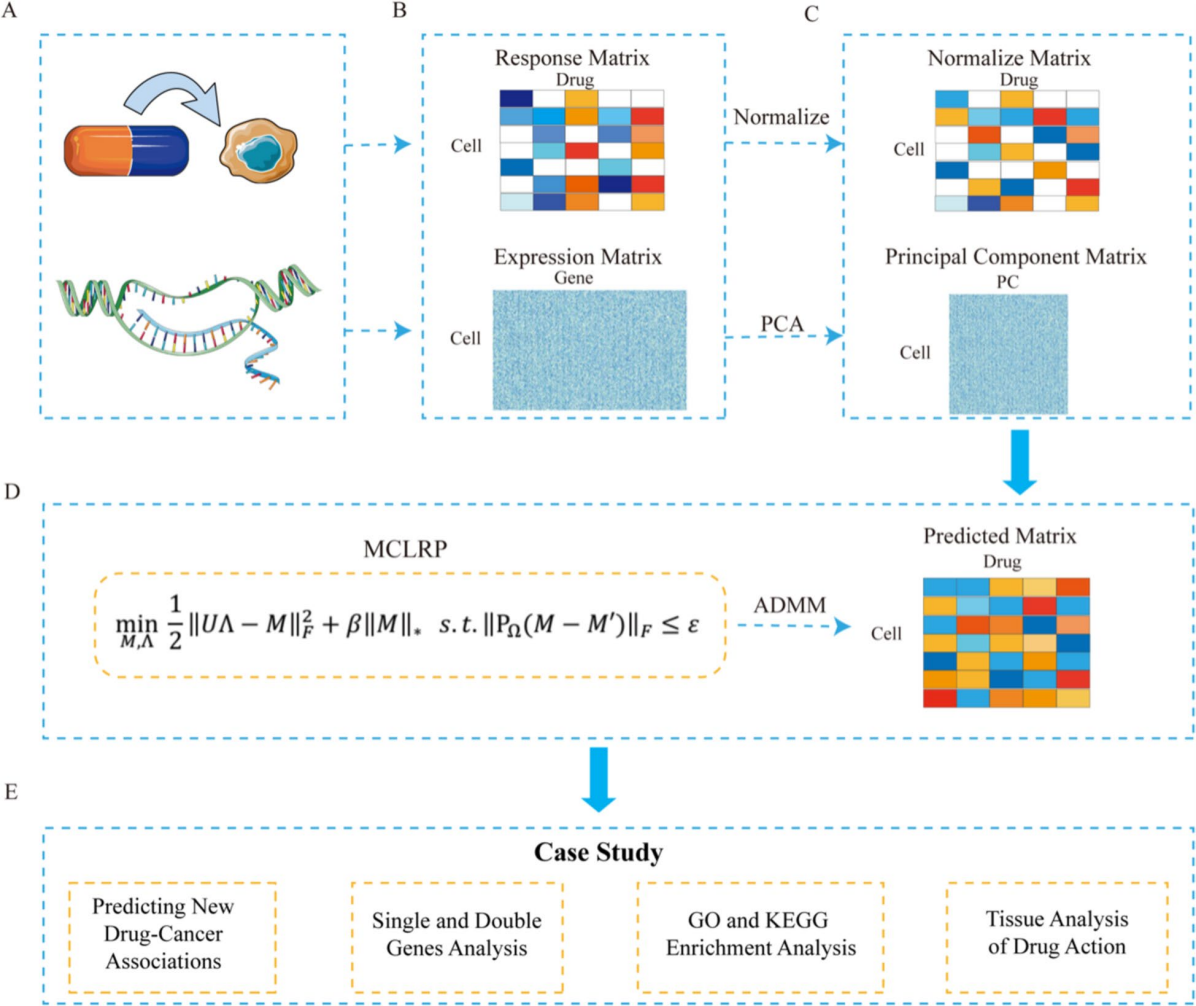
Flowchart illustrating MCLRP method for predicting anticancer drug response. **A** Generation of anticancer drug sensitivity and gene expression profiling data. **B** Collection of anticancer drug response and gene expression profiles. **C** Normalization of anticancer drug response and dimensionality reduction of gene expression profiles using principal component analysis. **D** The MCLRP model solves the problem by employing alternating direction method of multipliers (ADMM) to reconstruct the anticancer drug response. **E** Case study validating the predictions made by MCLRP through the prediction of new drug-cancer associations, analysis of single and co-mutations, GO and KEGG enrichment analyses, and tissue analysis of drug action

To leverage the available data effectively and uncover unknown drug responses, we employed a matrix completion model for predicting the response of anticancer drugs in cell lines. The workflow of the model is depicted in Fig. 8.

Several regression models, including ridge regression and elastic net, have demonstrated strong and consistent performance in various contexts [65]. In this study, we assumed a linear relationship between the gene-expression profiles of cell lines and the corresponding anticancer drug responses. However, given the large number of genes compared to the number of cell lines in our dataset, there was inherent redundancy in the information. To address this issue and prevent overfitting, we employed dimensionality reduction techniques on the gene expression profiles. Additional file 2: Table S14 illustrates the conditions of use of dimensionality reduction methods. PCA is a widely used unsupervised learning method that utilizes orthogonal transformations to convert the original data, characterized by linearly correlated variables, into a reduced set of variables that are linearly uncorrelated. Given the high dimensionality and redundancy of gene expression data (thousands of genes vs. hundreds of samples), we applied PCA to extract the major variance components. These components often correspond to biologically meaningful patterns such as pathways and cellular processes, allowing the model to retain essential biological signals while reducing noise and computational complexity.

We replace the missing values in the observed cell line-drug response matrix with 0, and then standardize each column to derive the normalized matrix denoted by $M{^{\prime}}=[{M}_{ik}{^{\prime}}{]}_{m\times p}$, where $m$ is the number of cell lines, and p is the number of drugs. We use $X=[{X}_{ij}{]}_{m\times n}$ to denote the cell line-gene expression matrix, where $n$ is the number of genes. Let $\Omega$ be a set that contains the index pair $\left(i,j\right)$ of all known entries in $M{^{\prime}}$, i.e., $M{{^{\prime}}}_{ij}\ne 0$, if $(i,j)\in \Omega$. We assume that the underlying real drug response matrix $M$ can be expressed linearly by the column of $X$ due to $\text{m}\ll \text{n}$. When measuring the drug sensitivity data, the model should effectively tolerate the potential noise due to the amount of noise that can be present in drug sensitivity data. The estimation and optimization problem of the drug response matrix is as follows:

$$\underset{M}{\text{min}}\ \text{rank}(M),\ \text{s.t.}\ \parallel P_{\Omega}(M - M') \parallel_{F} \le \varepsilon,\ M \in range(X)$$

where $\varepsilon$ is the allowable noise range between the final matrix $M$ and the initial matrix ${M}^{{^{\prime}}}$ in the known values, and ${P}_{\Omega }$ is the projection operator onto ${\Omega }$. Because the $rank(\cdot )$ function is a non-convex and discontinuous function, we use $\parallel \cdot {\parallel }_{*}$ to approximate $rank(\cdot )$, which could be formulated as:

$$\underset{M}{\text{min}}\, \parallel M{\parallel }_{*},\ \text{s.t.}\ \parallel {P}_{\Omega }\left(M-{M}^{\prime}\right){\parallel }_{F}\le \varepsilon,\ M\in range\left(X\right),$$

where $\parallel M{\parallel }_{\mathrm{*}}$ denotes the nuclear norm $M$, which is the sum of all singular values of M,. Now, the model is a convex optimization problem. Candès et al. proved that under certain conditions, the solution obtained by optimizing the nuclear norm is equivalent to that obtained by rank minimizing [66]. Because the dimension of the cell line-gene expression matrix $X$ is relatively high and has a significant redundancy of information, we use principal component analysis to reduce the dimension of $X$, which could be formulated as:

1 $$\underset{M, \Lambda }{\text{min}} \frac{1}{2}\parallel U\Lambda -M{\parallel }_{F}^{2}+\beta \parallel M{\parallel }_{*}, s.t.\parallel {P}_{\Omega }(M-M^{\prime}){\parallel }_{F}\le \varepsilon,$$

where $U=\text{PCA}\left(X\right)$, $\text{PCA}(\cdot)$ refers to principal component analysis of the gene expression profile $X$, and $\beta$ is the balance parameter.

### The MCLRP model

Due to the unspecified noise level, selecting appropriate parameters for the model becomes challenging. Additionally, developing an efficient solver for the conditional constraint model is not a straightforward task. To simplify the solving process, we relaxed the original model (1) to a regularized model. By introducing a soft regularization term, we not only accommodate unknown noise but also enhance computational convenience. The resulting unconstrained optimization problem is as follows:

2 $$\underset{M, \Lambda }{\text{min}} \frac{1}{2}\parallel U\Lambda -M{\parallel }_{F}^{2}+\beta \parallel M{\parallel }_{*}+{\delta }_{G}\left(M\right),$$

where $G=\{M:\parallel {P}_{\Omega }(M-M^{\prime}){\parallel }_{F}\le \varepsilon$. To facilitate the solution, we perform variable substitution on the optimization function (2), which is formulated as follows:

3 $$\underset{M, \Lambda }{\text{min}} \frac{1}{2}\parallel U\Lambda -M{\parallel }_{F}^{2}+\beta \parallel M{\parallel }_{*}+{\delta }_{G}\left(N\right), s.t.M=N.$$

Accordingly, the augmented Lagrangian method formula (3) is as follows:

4 $$\underset{M, \Lambda ,N,\Gamma }{\text{min}} \frac{1}{2}\parallel U\Lambda -M{\parallel }_{F}^{2}+\beta \parallel M{\parallel }_{*}+{\delta }_{G}(N)+\langle \Gamma ,M-N\rangle +\frac{\rho }{2}\parallel M-N{\parallel }_{F}^{2},$$

where $\Gamma$ is the Lagrange multiplier and $\rho>0$ is the penalty parameter. We solve the optimization problem (4) by using the alternating direction method of multipliers (ADMM), at the *k*th iteration,

5 $$\begin{array}{c}{\Lambda }^{k+1}=\text{arg}\;\underset{\Lambda }{\text{min}}\left\{\frac{1}{2}{\Vert U\Lambda -{M}^{k}\Vert }_{F}^{2}\right\}, \end{array}$$

6 $${M}^{k+1}=\text{arg}\;\underset{M}{\text{min}}\left\{\frac{1}{2}{\Vert U{\Lambda }^{k+1}-M\Vert }_{F}^{2}+\beta \parallel M{\parallel }_{*}+\frac{\rho }{2}\parallel M-{N}^{k}+{Y}^{k}{\parallel }_{F}^{2}\right\},$$

7 $${N}^{k+1}=\text{arg}\;\underset{N}{\text{min}}\left\{{\delta }_{G}\left(N\right)+\frac{\rho }{2}\parallel N-{M}^{k+1}-{Y}^{k}{\parallel }_{F}^{2}\right\},$$

8 $${Y}^{k+1}={Y}^{k}+{M}^{k+1}-{N}^{k+1} .$$

where ${Y}^{k}=\frac{1}{\rho }{\Gamma }^{k}$. For solving the problem (5), we obtain the partial derivative of the objective function, and then get $0 = U^{T}(U\Lambda - M^{k})$ and $U^{T} U \Lambda = U^{T} M^{k}$. Then, we obtained the following equation:

9 $${\Lambda }^{k+1}=({U}^{T}U{)}^{-1}{U}^{T}{M}^{k}.$$

For solving the problem (6), we obtain the partial derivative of the objective function, and begin to compute (6),

$$0\in M-U{\Lambda }^{k+1}+{\partial }_{{\beta \Vert \cdot \Vert }_{*}}\left(M\right)+\rho M-\rho {N}^{k}+\rho {Y}^{k}$$

$$0\in \left(1+\rho \right)M-\left(U{\Lambda }^{k+1}+\rho {N}^{k}-\rho {Y}^{k}\right)+{\partial }_{{\beta \Vert \cdot \Vert }_{*}}\left(M\right)$$

$$-M+\frac{1}{1+\rho }\left(U{\Lambda }^{k+1}+\rho {N}^{k}-\rho {Y}^{k}\right)\in {\partial }_{{\frac{\beta }{1+\rho }\Vert \cdot \Vert }_{*}}\left(M\right)$$

Finally, we obtain the following:

10 $${M}^{k+1}={prox}_{\frac{\beta }{1+\rho }\parallel \cdot {\parallel }_{*}}\left(\frac{1}{1+\rho }\left(U{\Lambda }^{k+1}+\rho {N}^{k}-\rho {Y}^{k}\right)\right),$$

where ${prox}_{\tau }\left(\cdot\right)$ is the singular value shrinkage operator. We use the projected gradient method to solve the problem (7). First, let $f(N)=\frac{\rho }{2}\parallel N-({M}^{k}+{Y}^{k}){\parallel }_{F}^{2}$, and $H^{k} = N^{k} - t_{k} \nabla f(N^{k})$. Then, the following formula can be derived from the projected gradient method.

11 $${N}^{k+1}={P}_{G}\left({H}^{k}\right)=\left\{\begin{array}{cc}{H}^{k}& ,{H}^{k}\in G\\ {P}_{{\Omega }^{C}}\left({H}^{k}\right)+\left[\frac{{P}_{\Omega }\left({H}^{k}-{M}^{{^{\prime}}}\right)}{\parallel {P}_{\Omega }\left({H}^{k}-{M}^{{^{\prime}}}\right){\parallel }_{F}}\varepsilon +{P}_{\Omega }\left({M}^{{^{\prime}}}\right)\right]& ,{H}^{k}\notin G\end{array}\right.,$$

where $${\Omega }_{ij}=\left\{\begin{array}{cc}1& if {M}_{ij}{^{\prime}}\ne 0\\ 0& if {M}_{ij}{^{\prime}}=0\end{array}\right. and \, {\delta }_{G}(M)=\left\{\begin{array}{cc}0& if M\in G\\ +\infty & if M\notin G\end{array}\right.$$

The hyperparameters $\upbeta$ and $\uprho$ in (5–8) were optimized using a grid search method. This approach systematically tests different parameter combinations within a pre-defined range to identify the optimal values based on validation set performance. Importantly, these hyperparameters were kept consistent across all datasets, ensuring fairness and stability during model validation.

### Performance metrics

We performed tenfold cross-validation to assess the performance of the proposed models. To enhance the reliability of the results, we repeated the entire cross-validation process ten times to estimate the average performance of the model. To evaluate the predictive ability of our model for cell line drug response, we computed the Pearson correlation coefficient (PCC) and Spearman correlation coefficient (SCC) for each drug, comparing the predicted response data with the observed values.

The calculation of PCC is formulated as follows:

$$\text{PCC} = \frac{\sum_{i=1}^{k} (r_{i} - \bar{r}) (\hat{r}_{i} - \bar{\hat{r}}) }{ \sqrt{\sum_{i=1}^{k} (r_{i} - \bar{r})^{2} \sum_{i=1}^{k} (\hat{r}_{i} - \bar{\hat{r}})^{2}} }$$

where $\text{r}$ and $\widehat{\text{r}}$ represent the original and predicted response values, respectively. The average values are denoted by $\overline{r }$ and $\bar{\widehat{r}}$.$\text{k}$ is the number of known response values for each drug. The larger the PCC value, the more accurate the prediction.

The SCC for a drug could be calculated as

$${\text{SCC}}=1-\frac{6\sum_{i=1}^{k}{d}_{i}^{2}}{k\left({k}^{2}-1\right)},$$

where ${d}_{i}$ represents the rank of the $i$ thvalue, and $k$ is the number of known response values for each drug.

### Downstream biological analyses

After completing drug response prediction with MCLRP, we conducted multiple downstream analyses to validate and interpret the model outputs from a biological perspective. These analyses included:

(i) prediction of novel drug–cancer associations based on the top-ranked sensitivity scores for each drug; (ii) evaluation of drug response differences in cell lines harboring single or co-occurring gene mutations; (iii) construction of drug–gene association networks to investigate mutation–drug relationships; (iv) pathway enrichment analysis of genes associated with drug sensitivity; and (v) tissue-level response analysis to explore tissue-specific drug sensitivity patterns. Detailed procedures for each analysis are provided in the “Results” section.

## Supplementary Information

Additional file 1. Figure S1. Prediction performance by the comparison of the five models in GDSC dataset, evaluated by PCC. Figure S2. Prediction performance by the comparison of the five models in GDSC dataset, evaluated by SCC. Figure S3. Prediction performance by the comparison of MCLRP, DeepIC50, and GeneVAE in the GDSC dataset. Figure S4. Comparative performance of MCLRP and its ablated variants on the GDSC dataset in terms of PCC and SCC.

Additional file 2. Tables S1-S4. The data for the predictive high sensitivity response values selected by different thresholds. Table S5. The data for the relationship between additional drugs and co-mutations on one or more genes. Tables S6-S9. The top 1000 genes corresponding to each drug in the five datasets. Table S10-S13. Functional enrichment analysis of the top 1000 genes for each drug and the terms with a Benjamin false discovery rate of < 0.05. Table S14. Conditions of use for the methods.

## Data Availability

All data generated or analyzed during this study are included in this published article, its supplementary information files, and publicly available repositories. The codes and the data sets for predicting drug response are publicly available from Zenodo (10.5281/zenodo.8127448) [67] and https://github.com/kunwangouc/MCLRP [68]. The analyzed drug response data were obtained from two widely used benchmark resources: the Cancer Cell Line Encyclopedia (CCLE, https://www.broadinstitute.org/ccle) [69] and the Genomics of Drug Sensitivity in Cancer project (GDSC, https://www.cancerrxgene.org) [70], which provide comprehensive genomic, transcriptomic, and drug response profiles across hundreds of cancer cell lines.

## Abbreviations

MCLRP: Matrix Completion with Low-rank Regularization and Principal Component Analysis
PCA: Principal component analysis
GDSC: Genomics of Drug Sensitivity in Cancer
CCLE: Cancer Cell Line Encyclopedia
SRMF: Similarity-regularized matrix factorization
MCRR: Matrix Completion with Ridge Regression
NN: Neural network
CNN: Convolutional neural network
FC-NN: Fully connected neural network
DRP: Drug response prediction
SMILES: Simplified Molecular Input Line Entry System
VAE: Variational autoencoder
GO: Gene Ontology
KEGG: Kyoto Encyclopedia of Genes and Genomes
MAPK: Mitogen-activated protein kinase
NSCLC: Non-small cell lung cancer
AUC: Area under the curve
IC50: Half maximal inhibitory concentration
mTOR: Mammalian target of rapamycin
ADMM: Alternating direction method of multipliers
PCC: Pearson correlation coefficient
SCC: Spearman correlation coefficient
XAI: Explainable artificial intelligence
