# Supplementary figures and images for: MCLRP: enhanced prediction of anticancer drug response through low-rank matrix completion and transcriptomic profiling
Source: BMC Biol. 2025 Dec 3;24:4. doi: 10.1186/s12915-025-02457-8 (PMC12781783; doi:10.1186/s12915-025-02457-8)

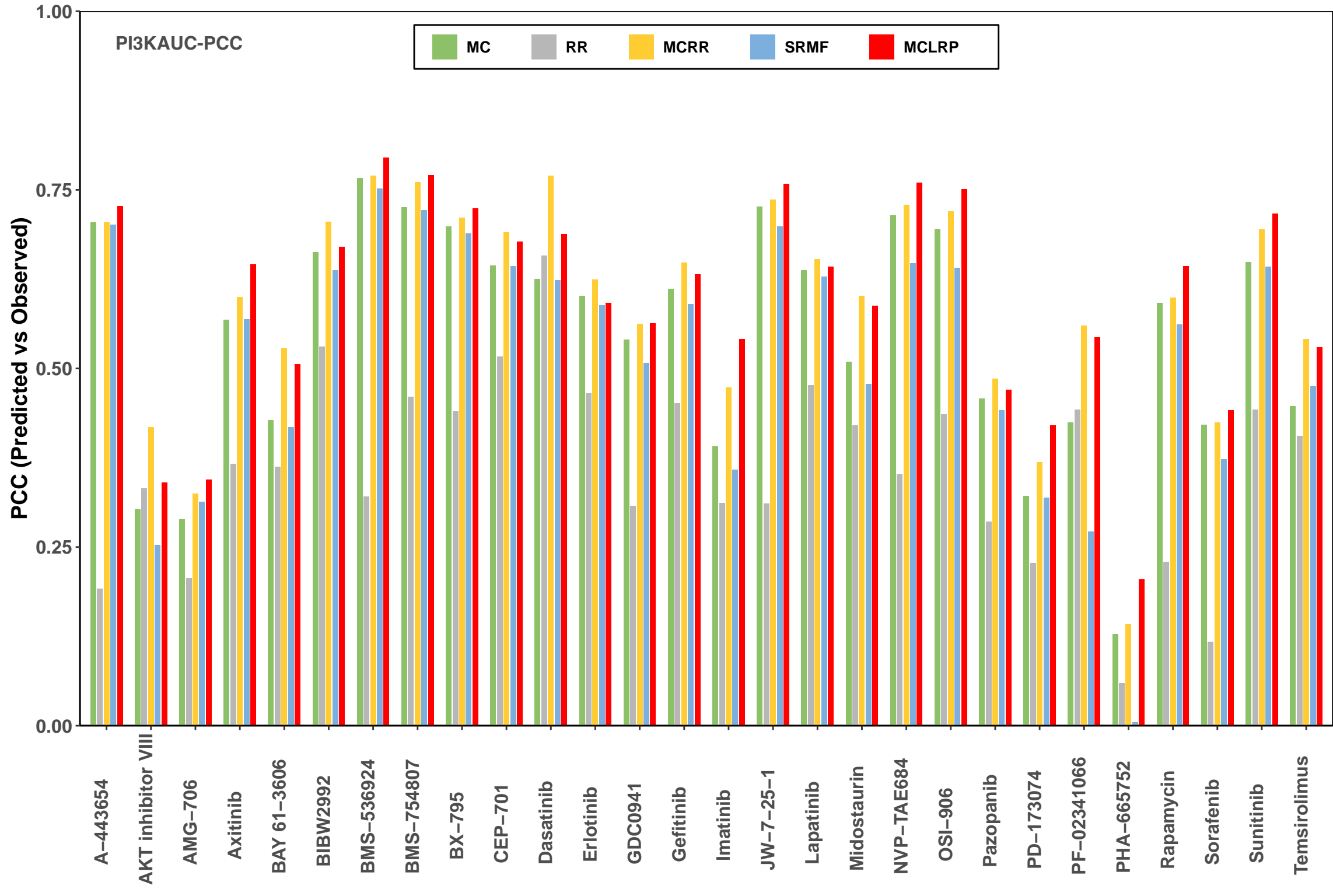

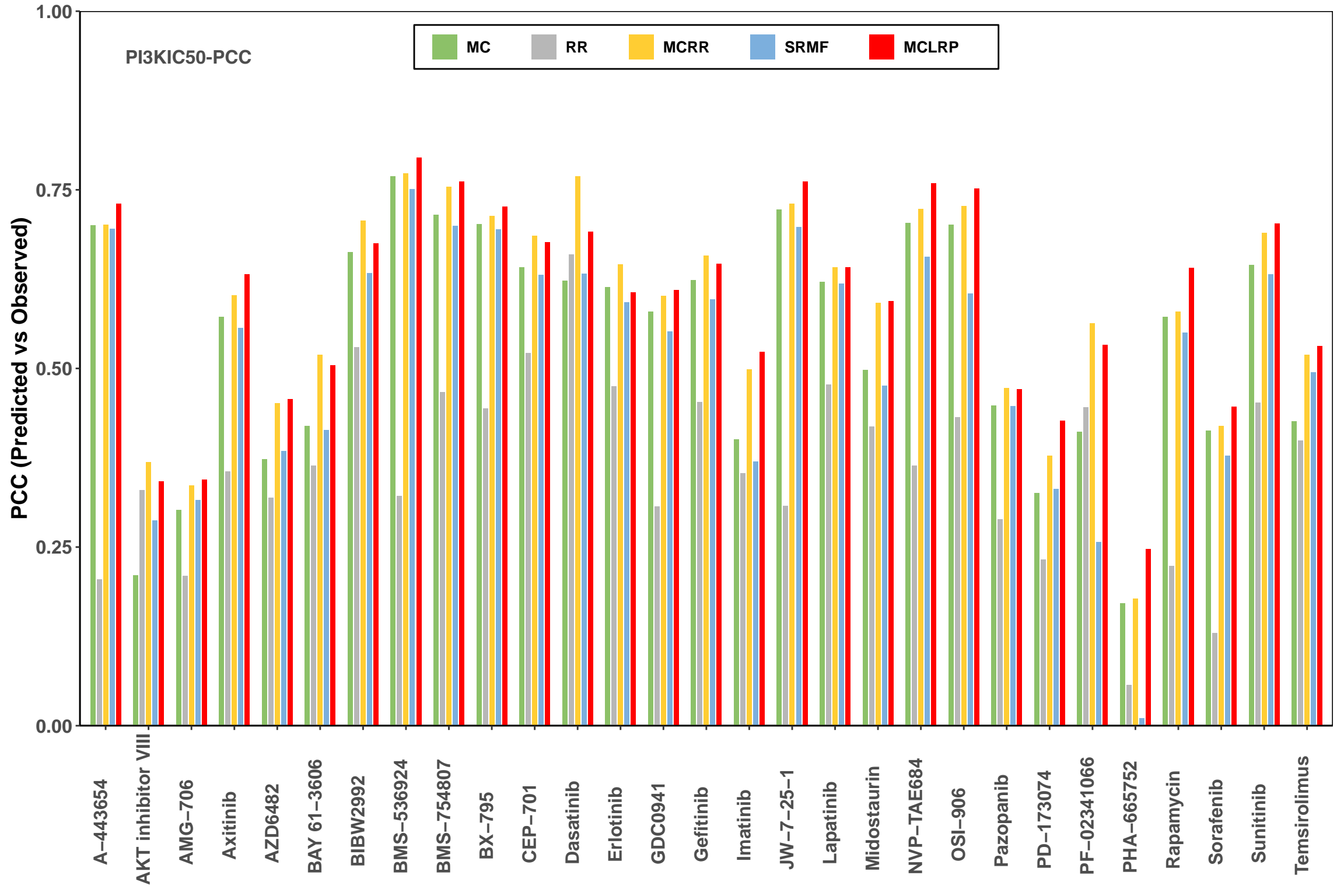

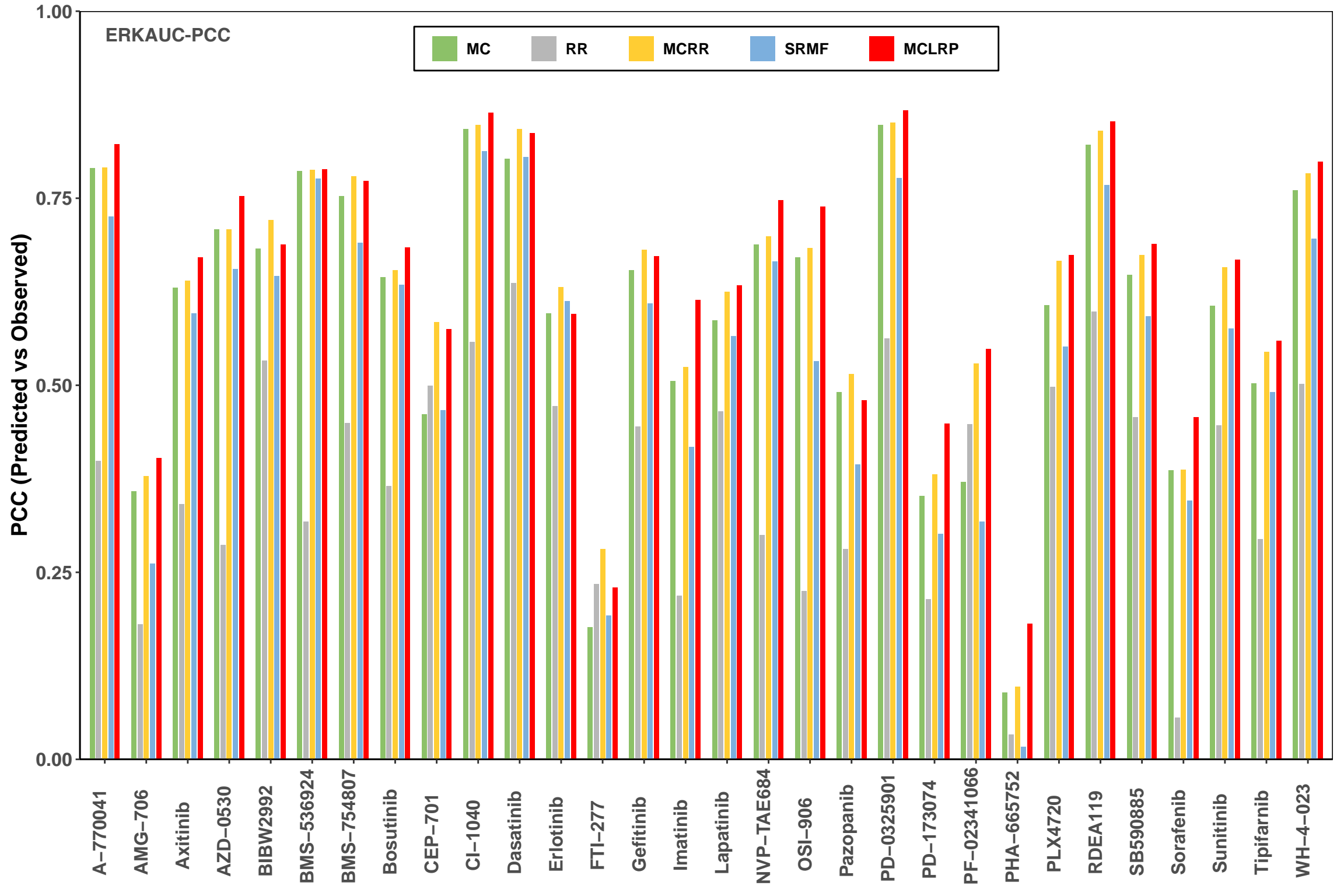

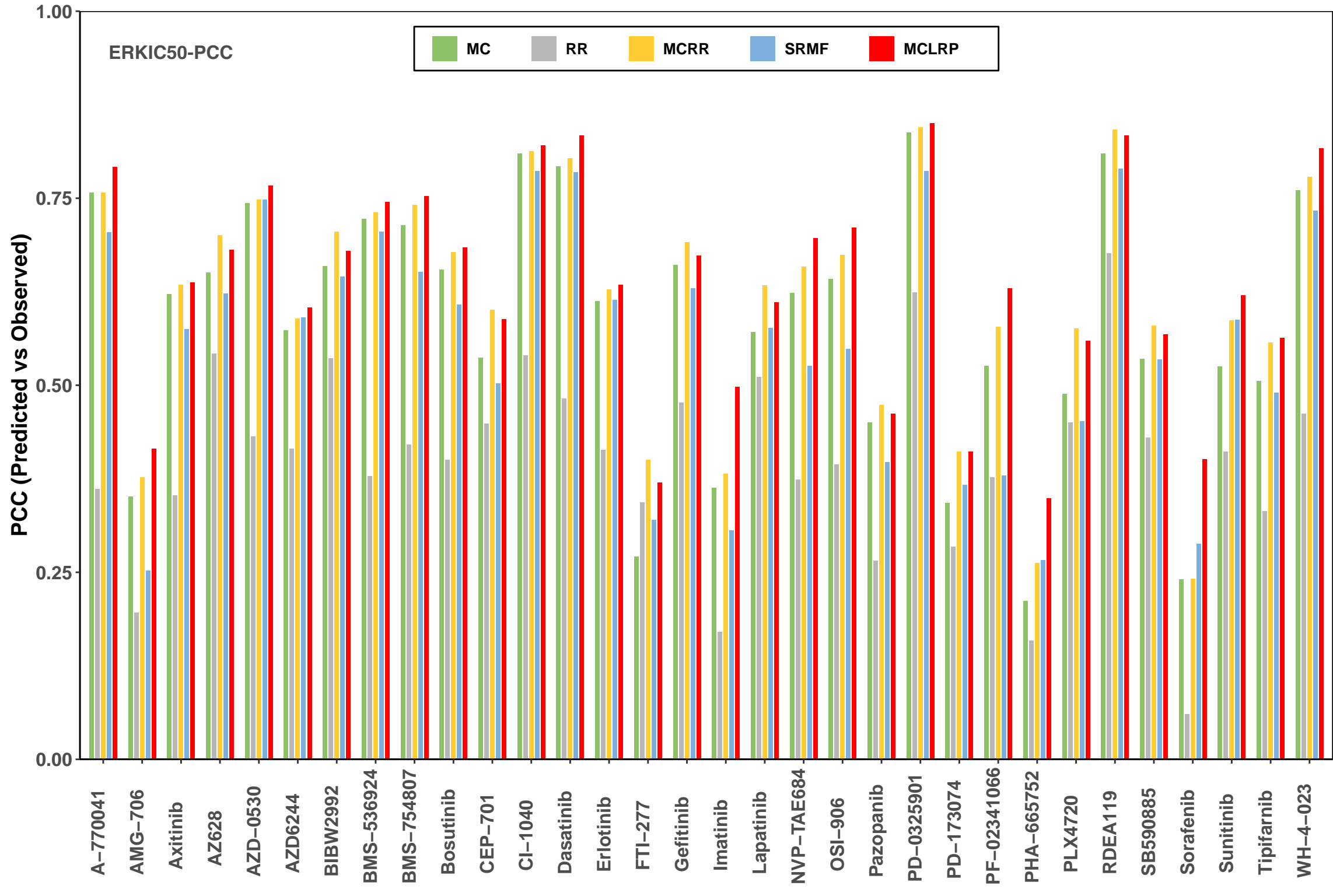

Supplement: Supplementary file 1 — Additional file 1. Figure S1. Prediction performance by the comparison of the five models in GDSC dataset, evaluated by PCC. Figure S2. Prediction performance by the comparison of the five models in GDSC dataset, evaluated by SCC. Figure S3. Prediction performance by the comparison of MCLRP, DeepIC50, and GeneVAE in the GDSC dataset. Figure S4. Comparative performance of MCLRP and its ablated variants on the GDSC dataset in terms of PCC and SCC. [file 12915_2025_2457_MOESM1_ESM.zip › Figure S1-PCC.pdf]

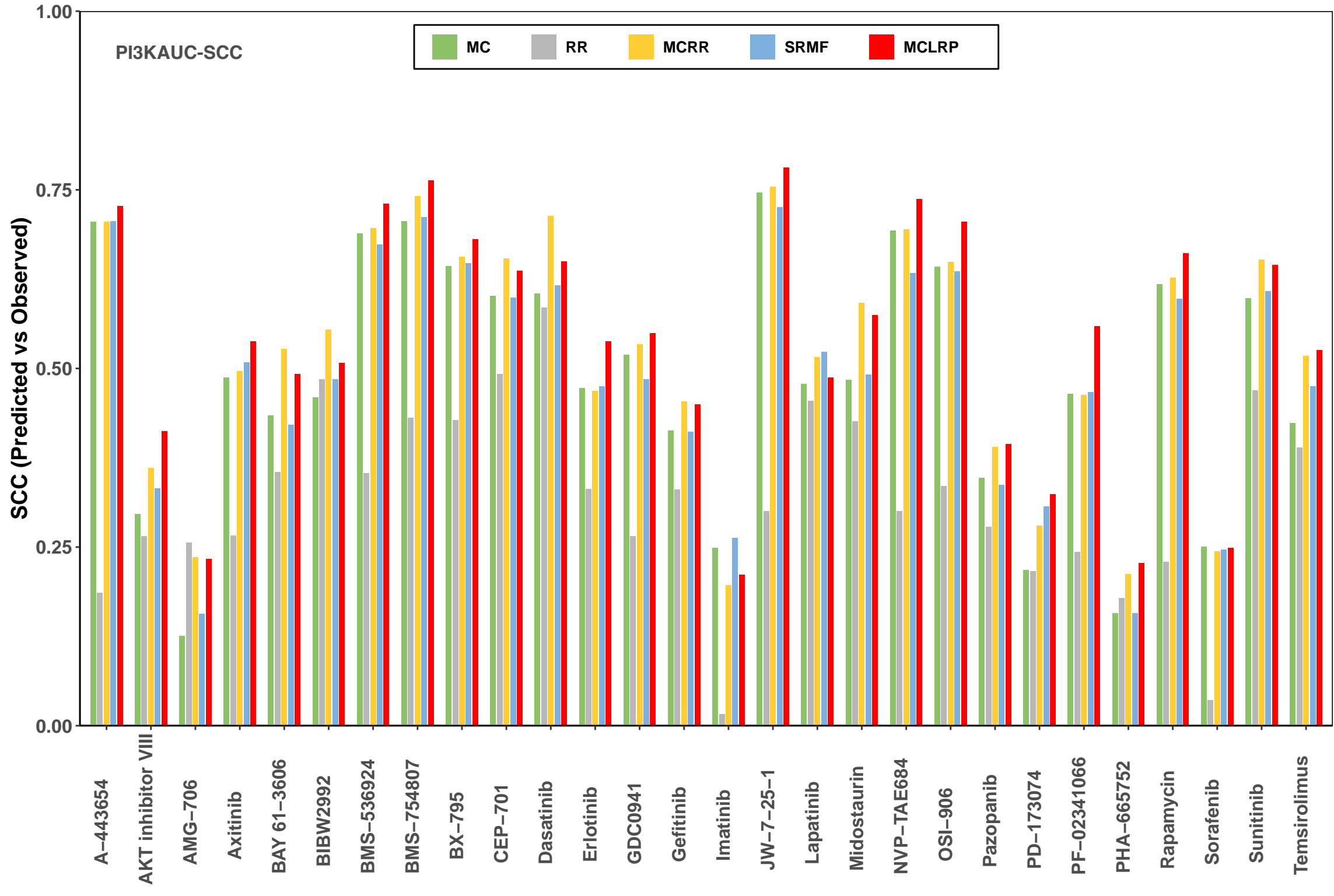

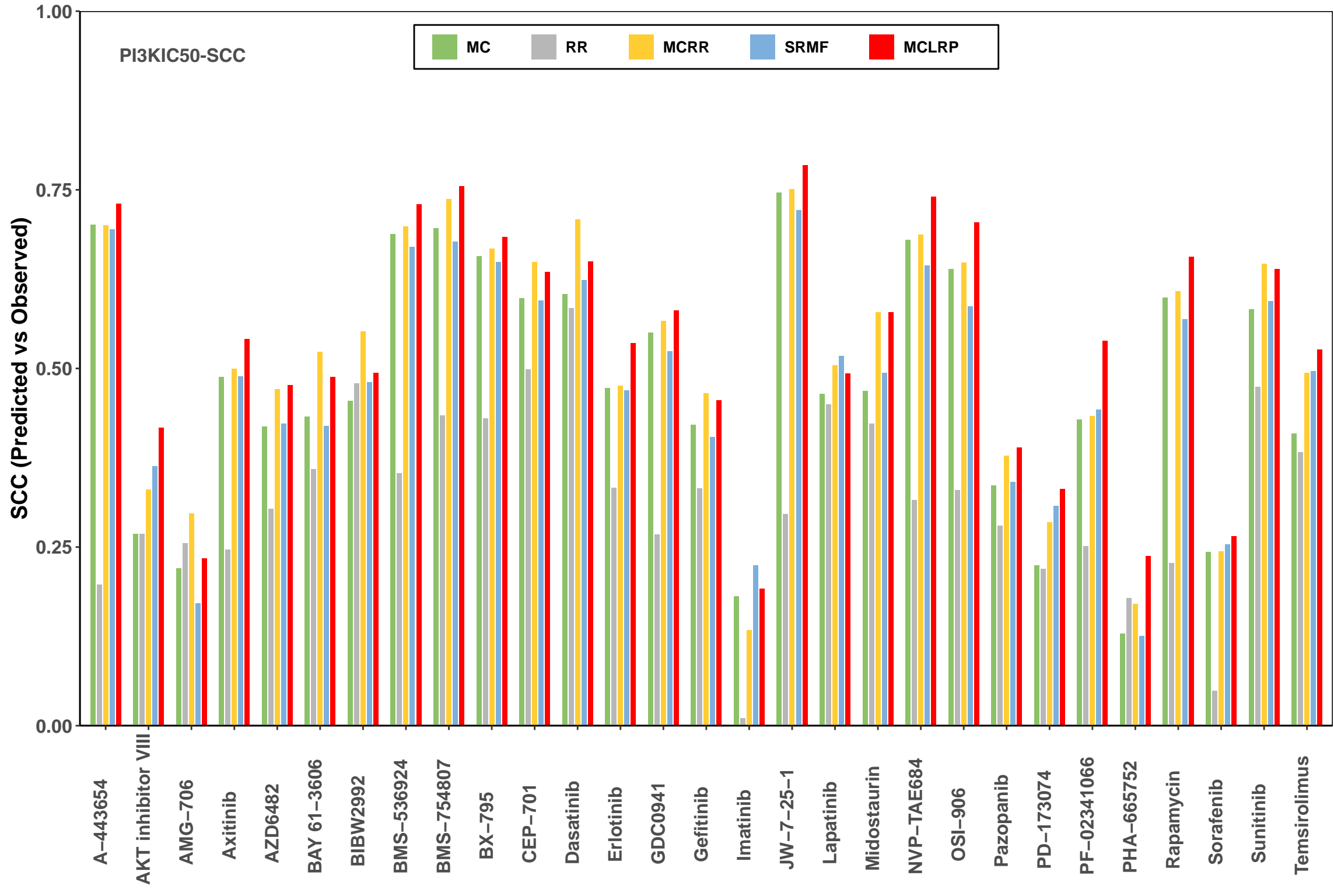

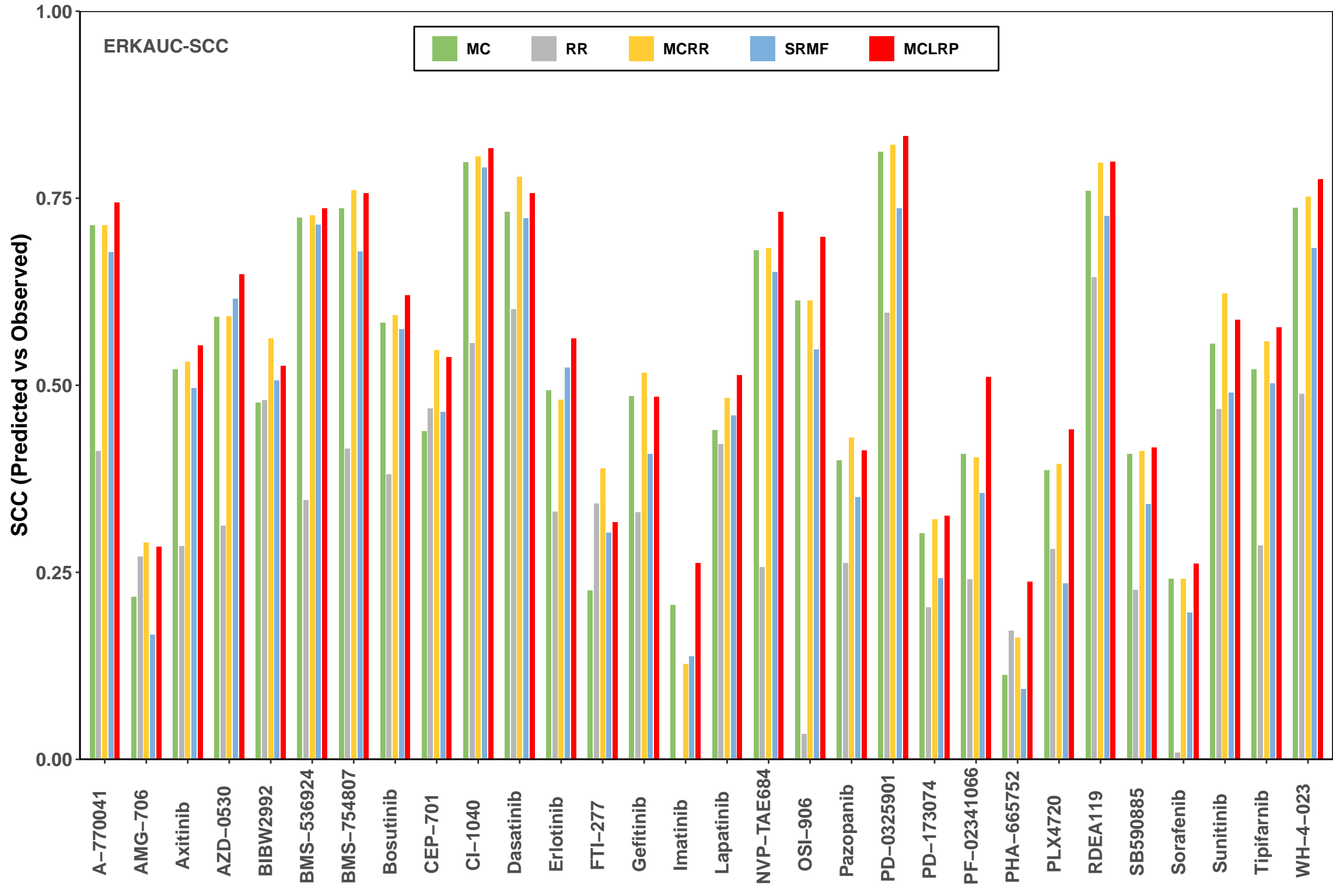

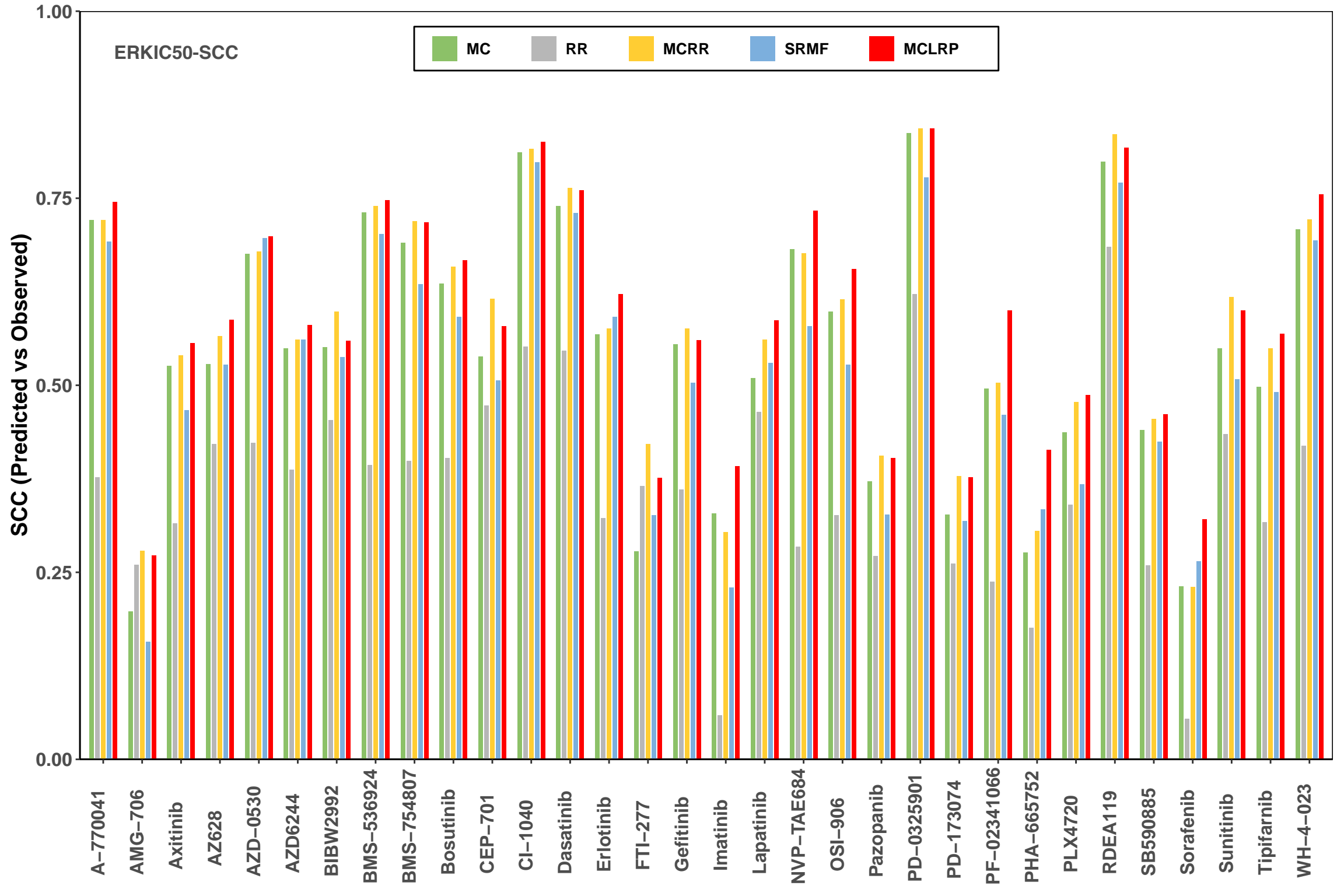

Supplement: Supplementary file 1 — Additional file 1. Figure S1. Prediction performance by the comparison of the five models in GDSC dataset, evaluated by PCC. Figure S2. Prediction performance by the comparison of the five models in GDSC dataset, evaluated by SCC. Figure S3. Prediction performance by the comparison of MCLRP, DeepIC50, and GeneVAE in the GDSC dataset. Figure S4. Comparative performance of MCLRP and its ablated variants on the GDSC dataset in terms of PCC and SCC. [file 12915_2025_2457_MOESM1_ESM.zip › Figure S2-SCC.pdf]

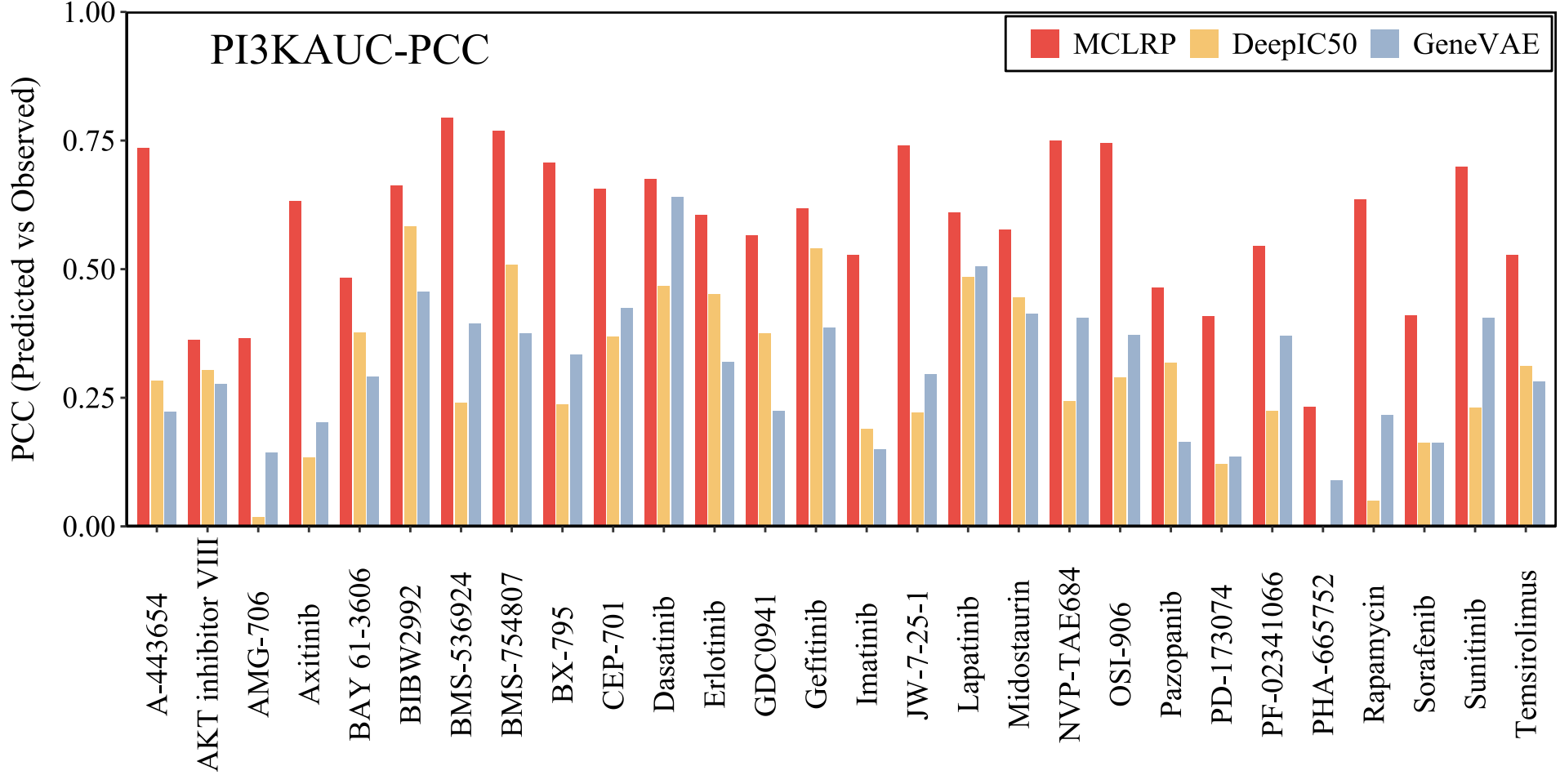

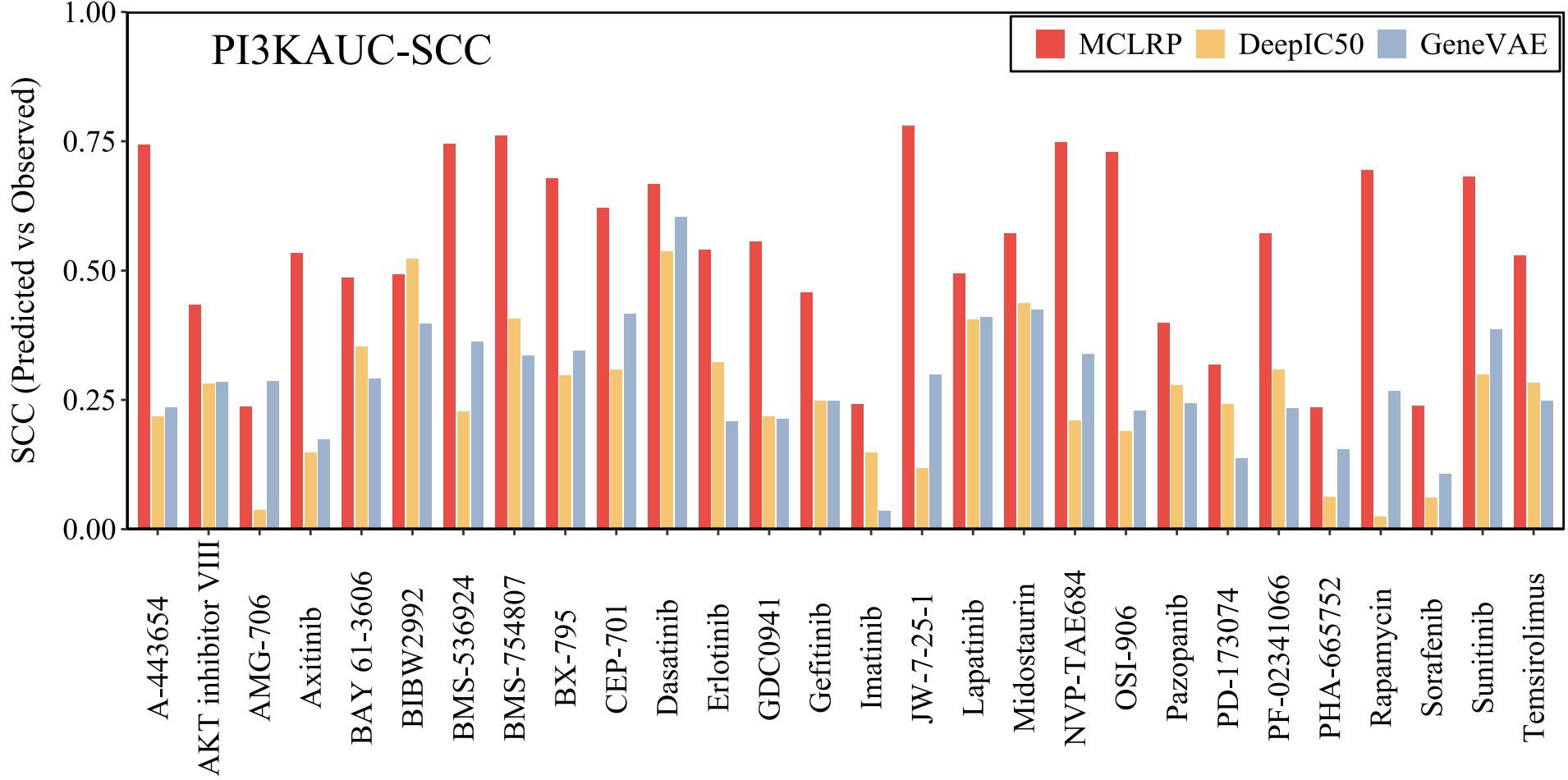

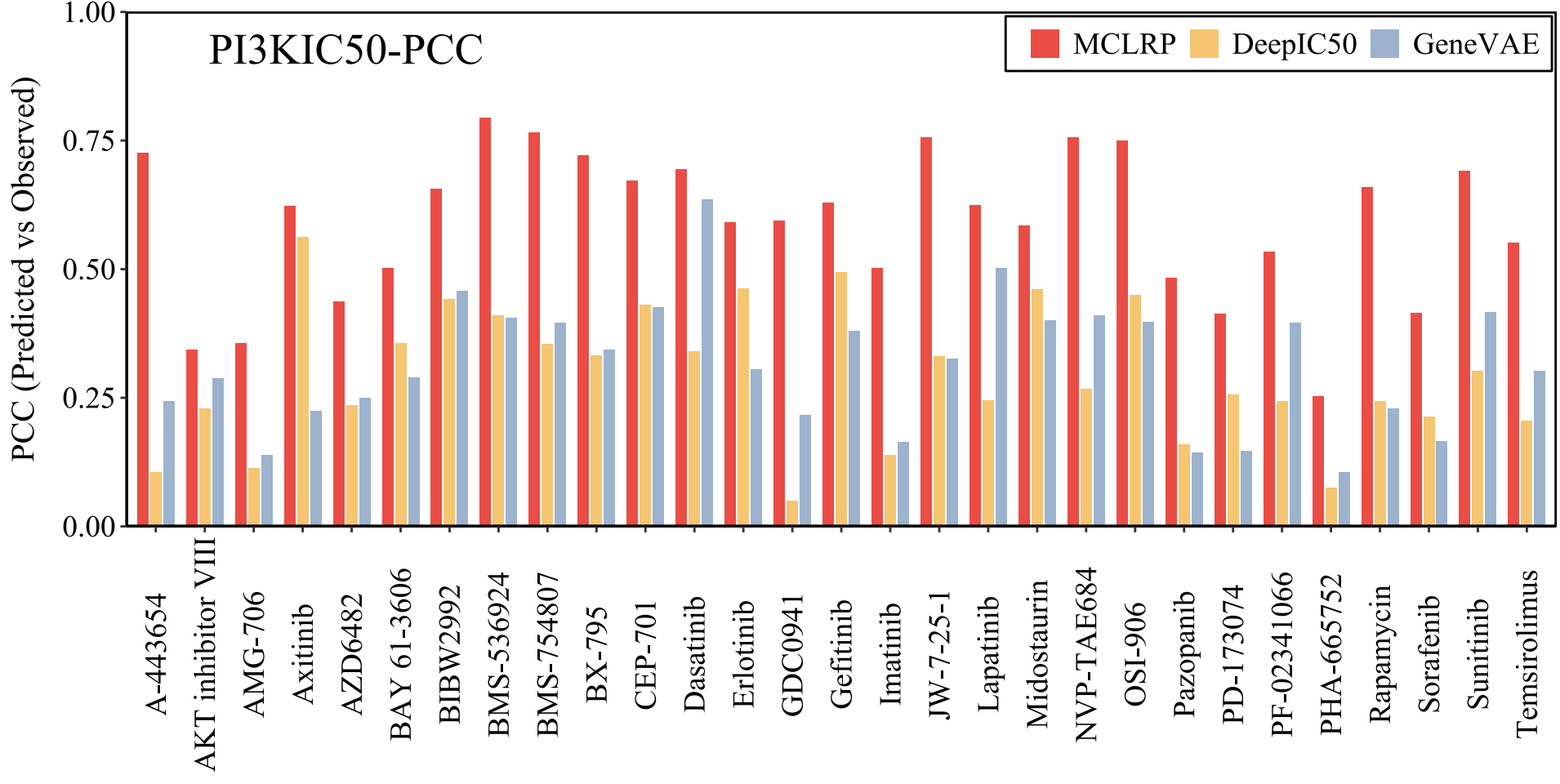

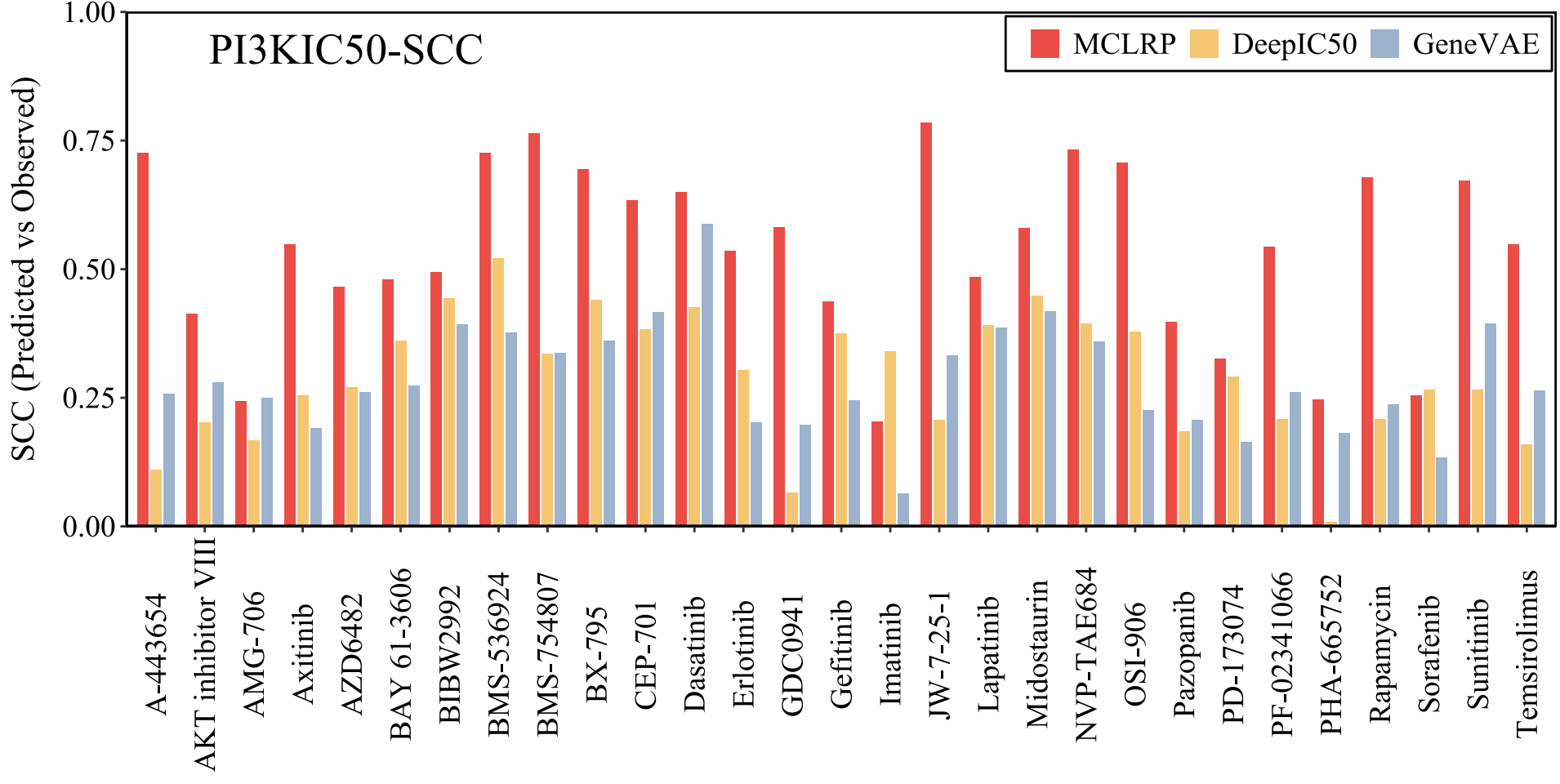

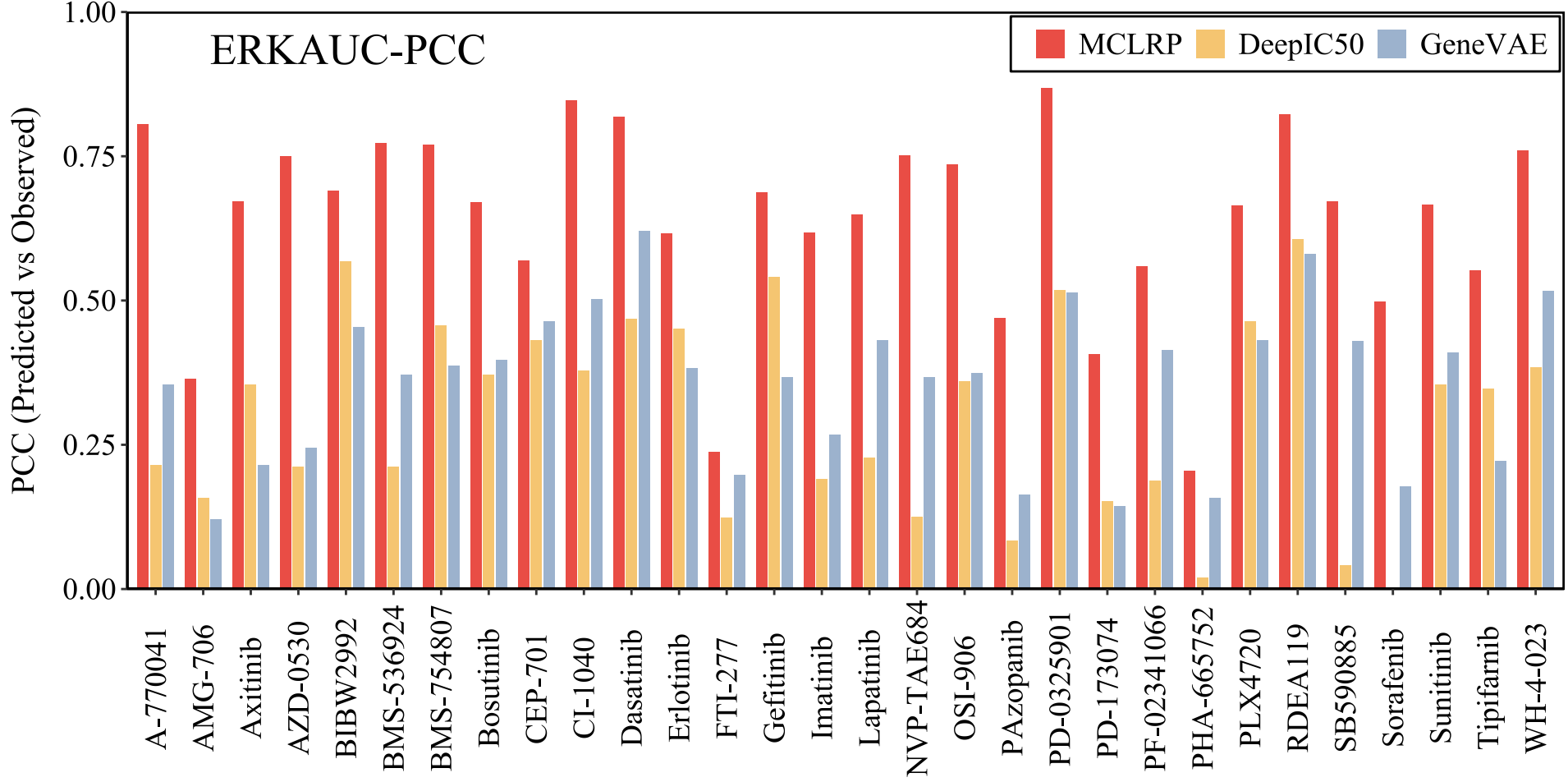

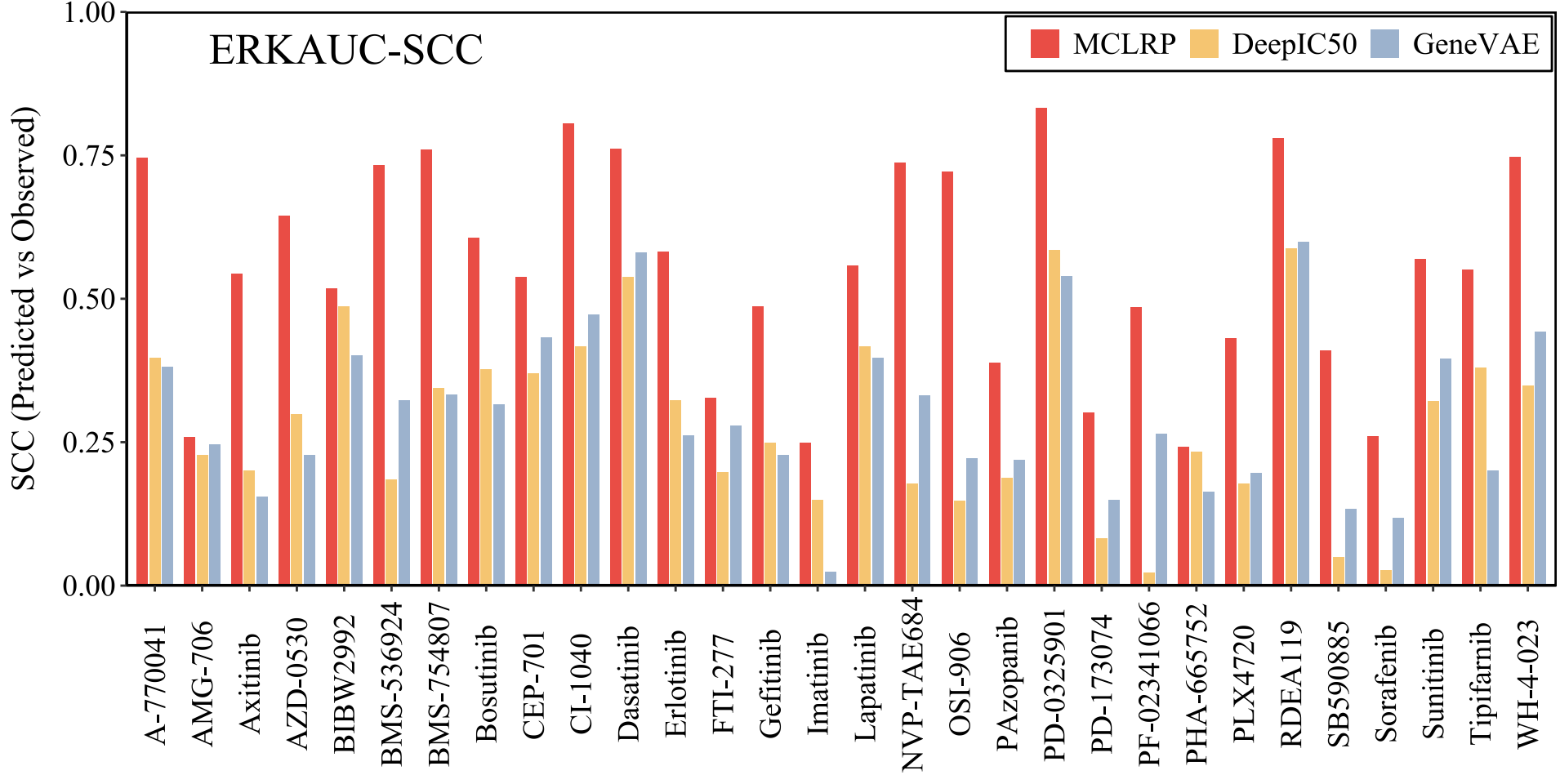

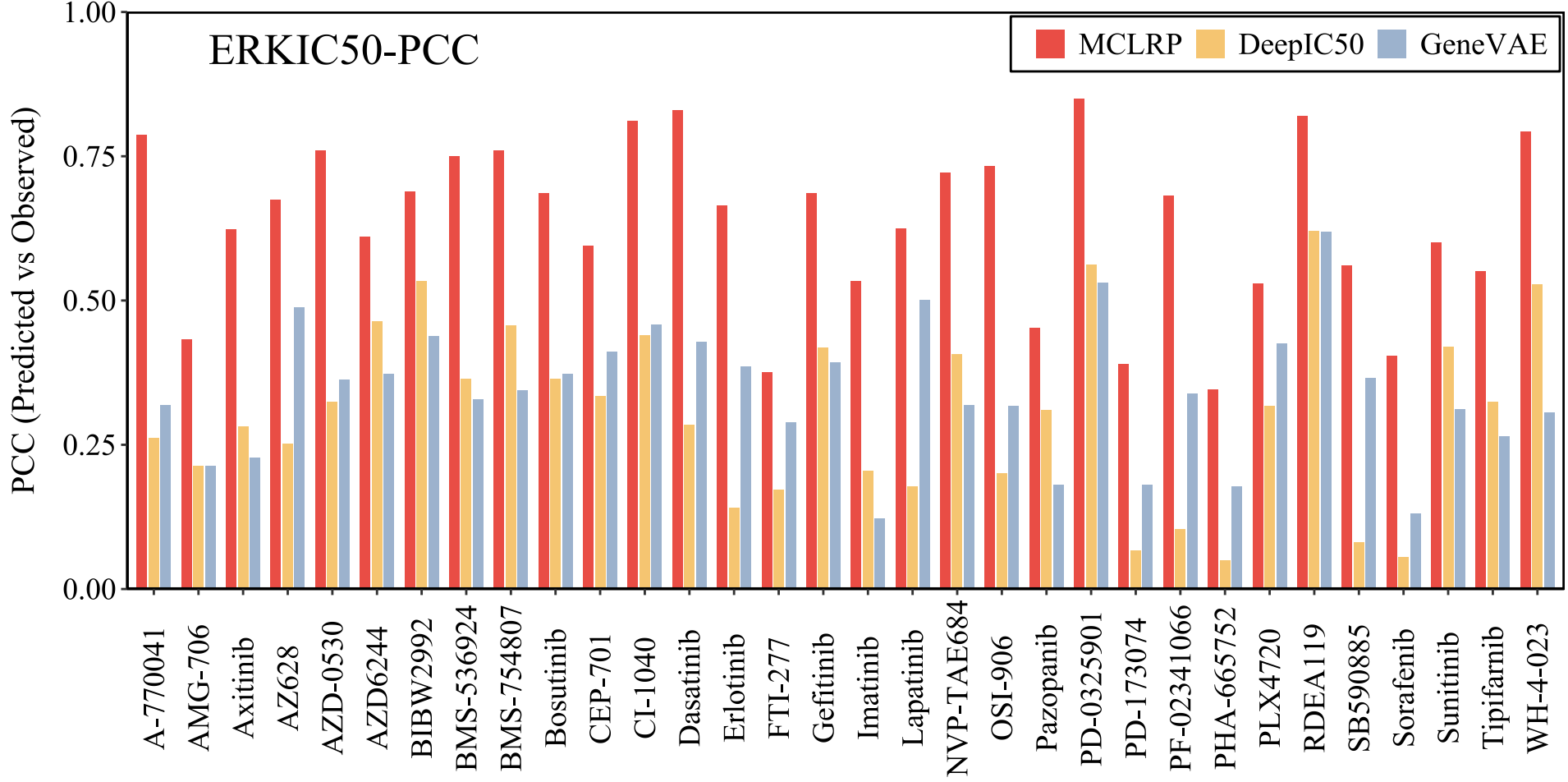

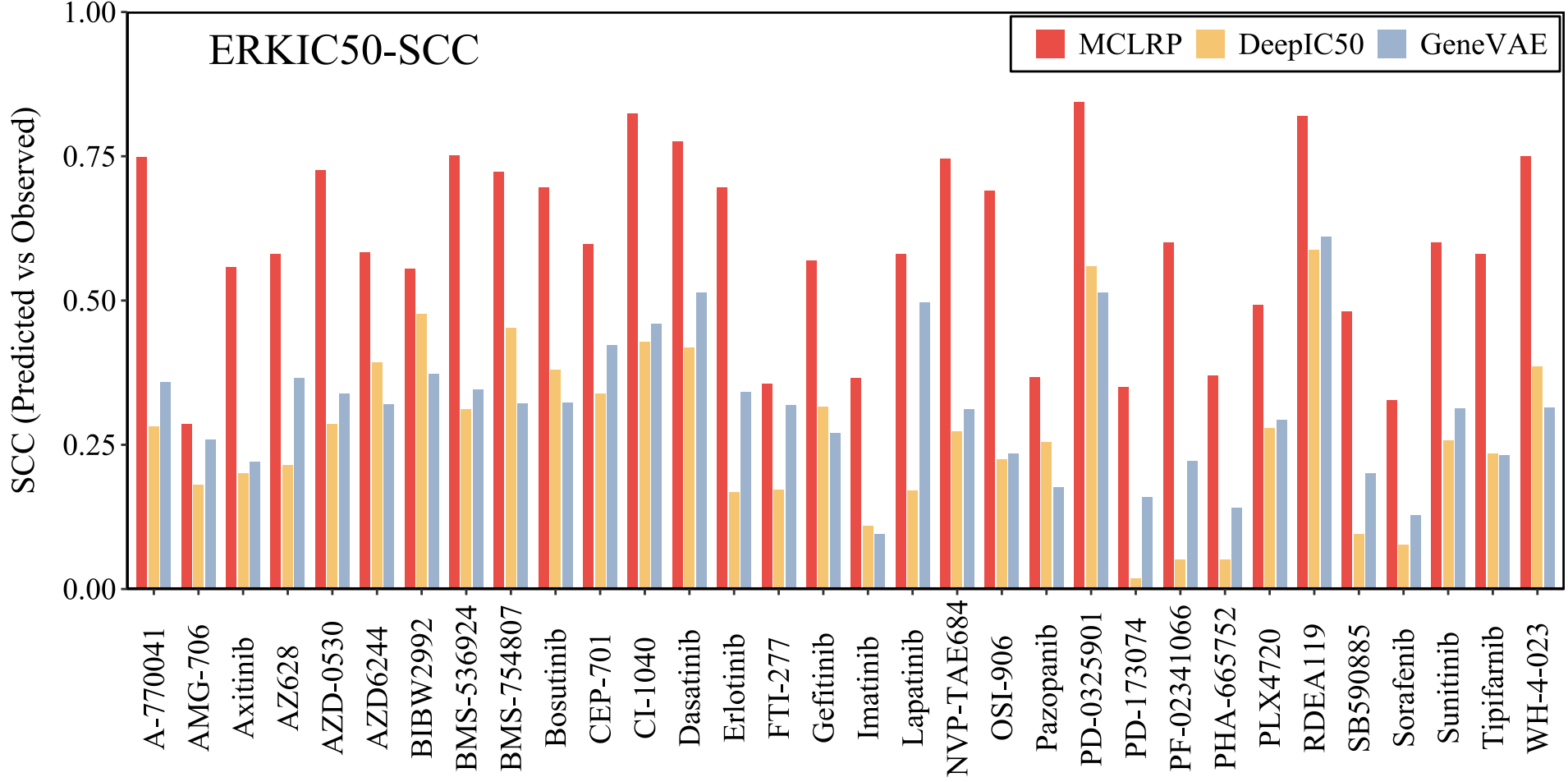

Supplement: Supplementary file 1 — Additional file 1. Figure S1. Prediction performance by the comparison of the five models in GDSC dataset, evaluated by PCC. Figure S2. Prediction performance by the comparison of the five models in GDSC dataset, evaluated by SCC. Figure S3. Prediction performance by the comparison of MCLRP, DeepIC50, and GeneVAE in the GDSC dataset. Figure S4. Comparative performance of MCLRP and its ablated variants on the GDSC dataset in terms of PCC and SCC. [file 12915_2025_2457_MOESM1_ESM.zip › Figure S3-Deep learning.pdf]

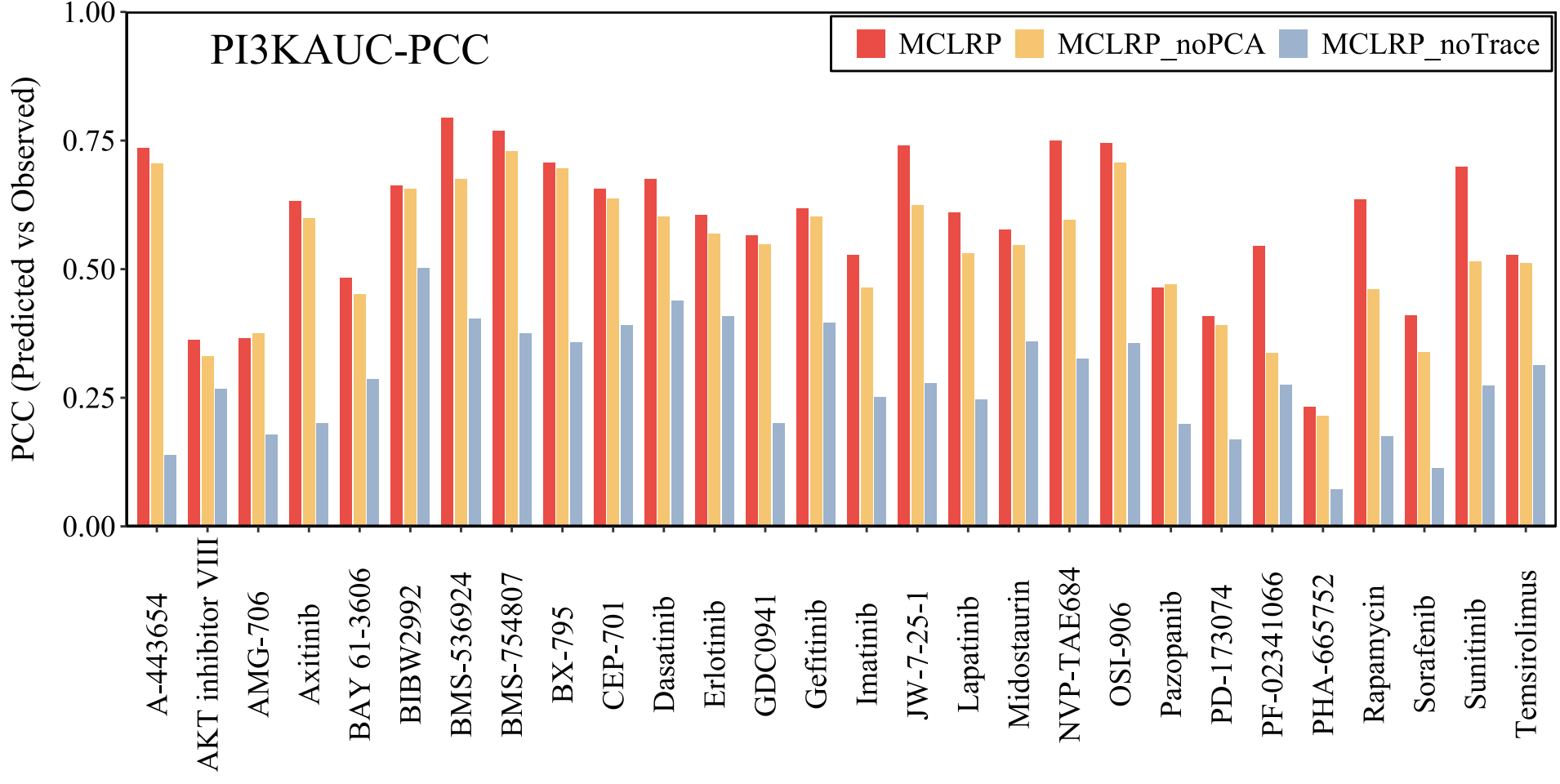

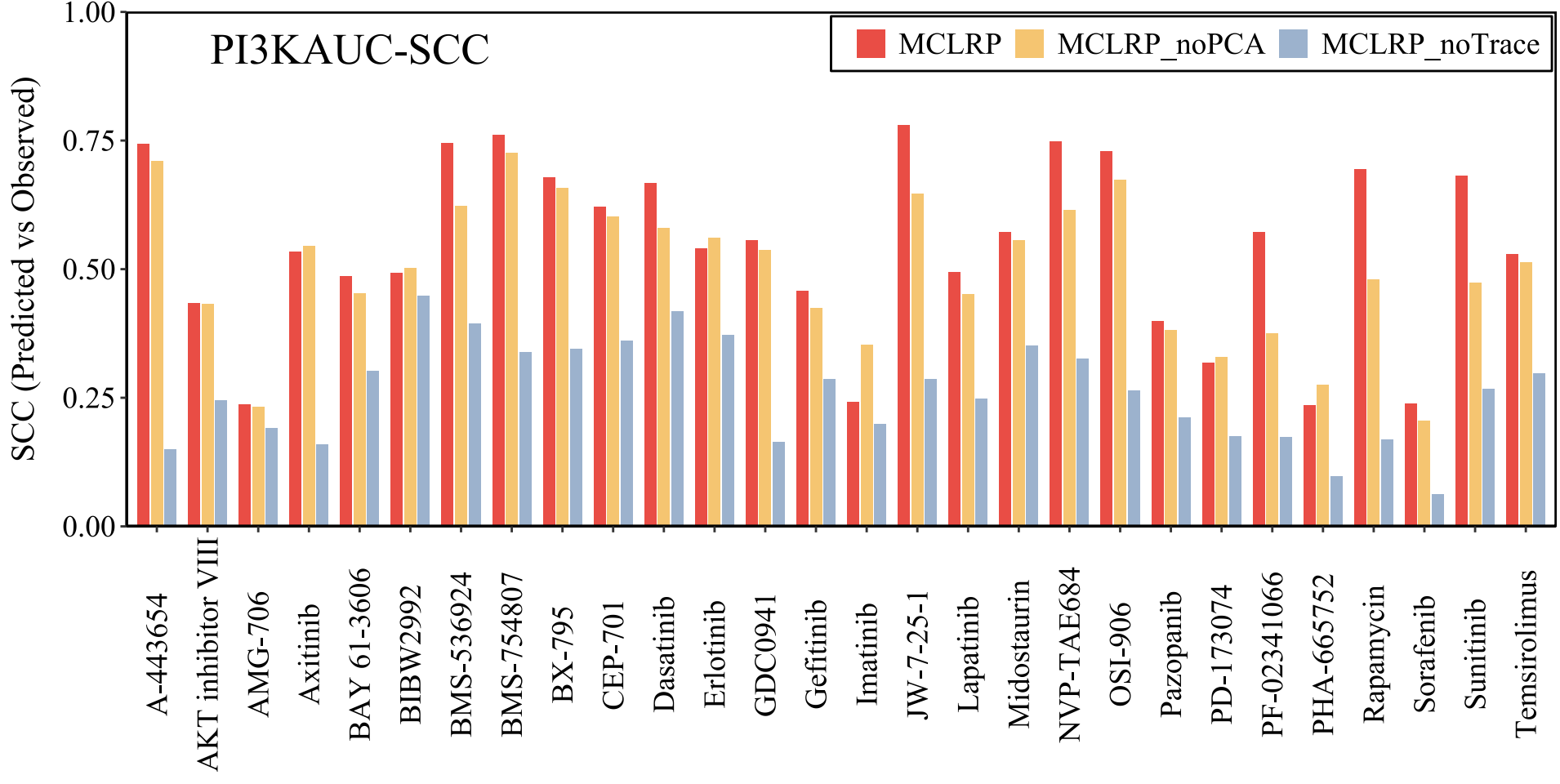

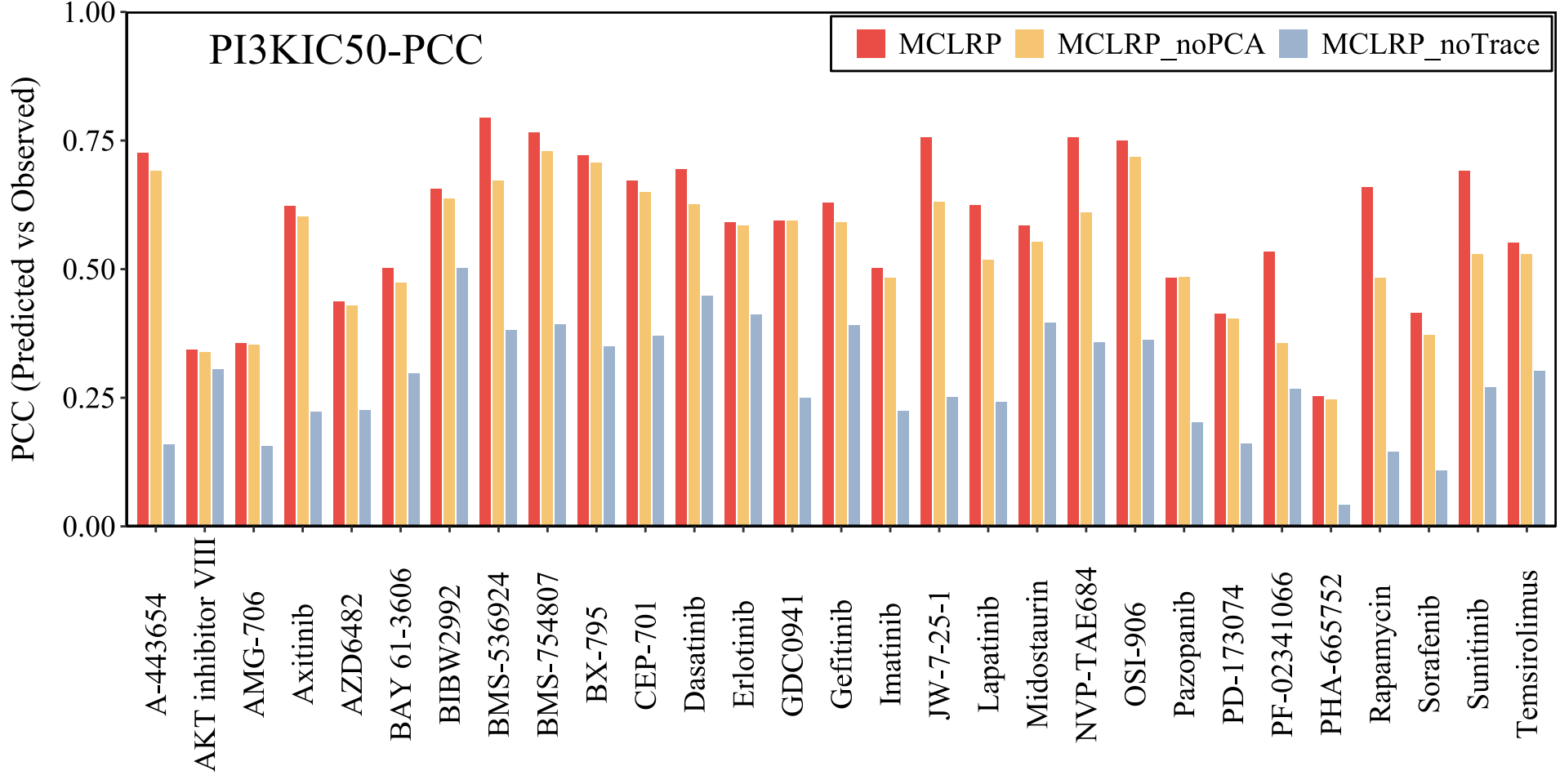

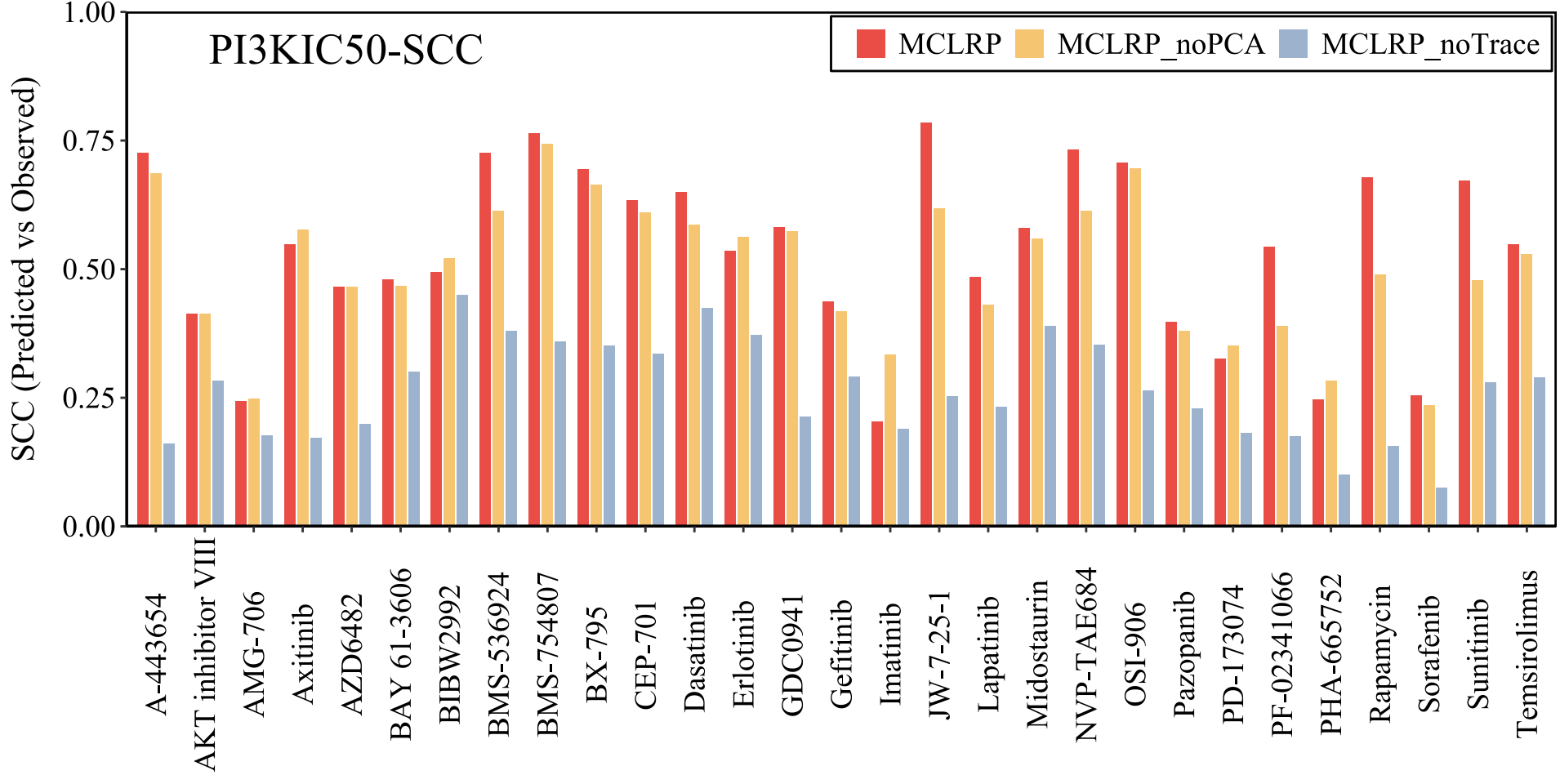

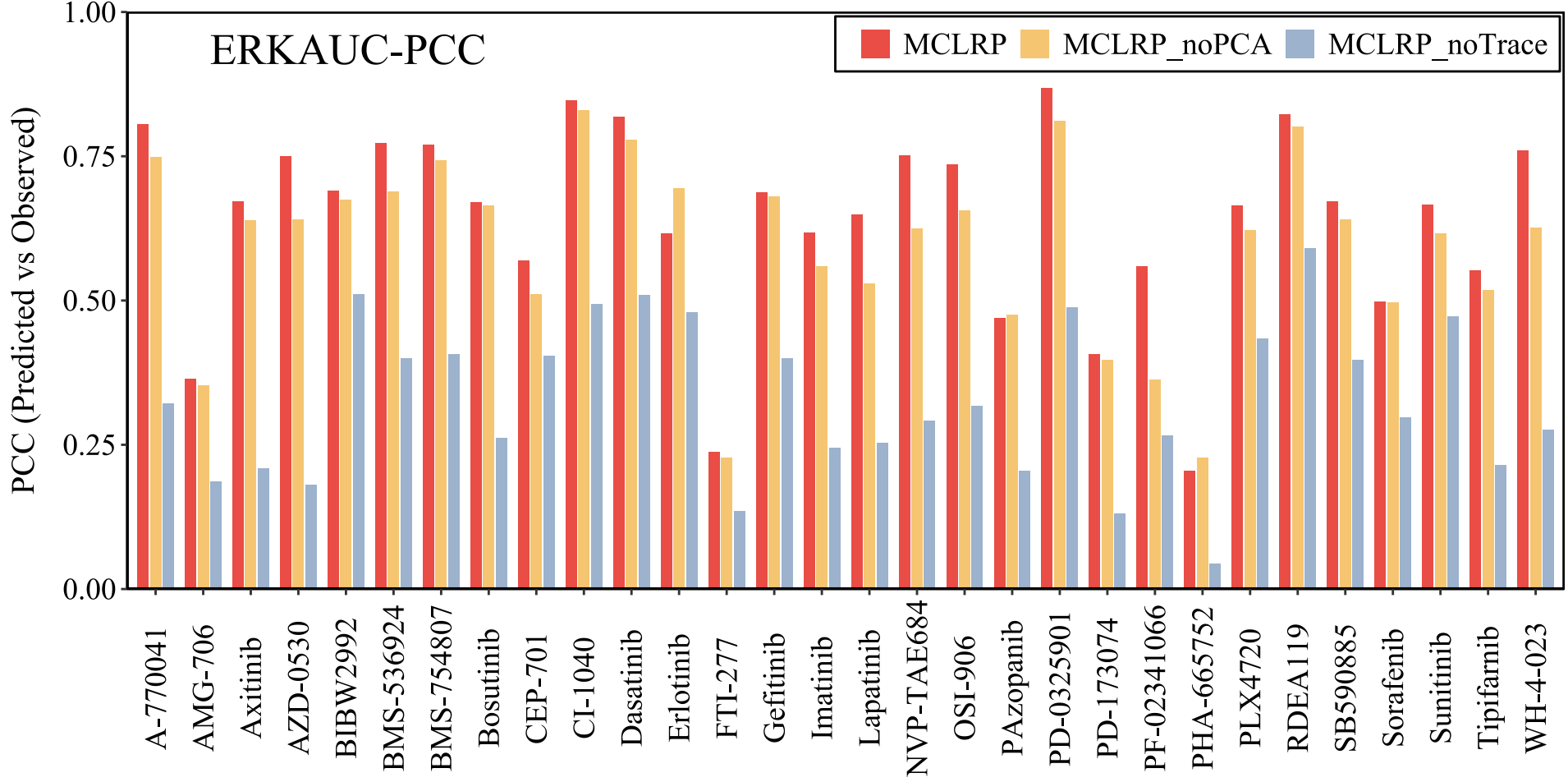

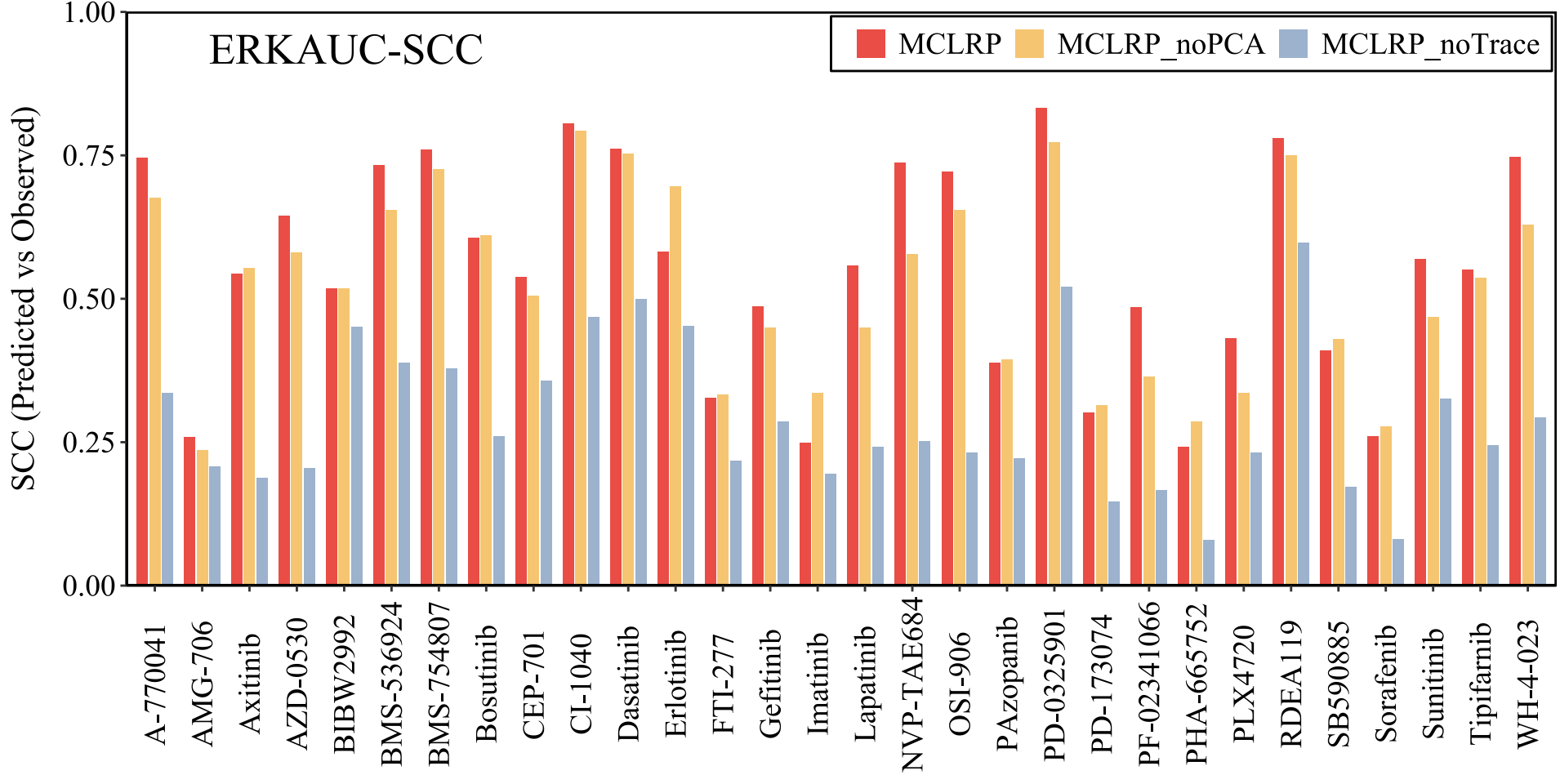

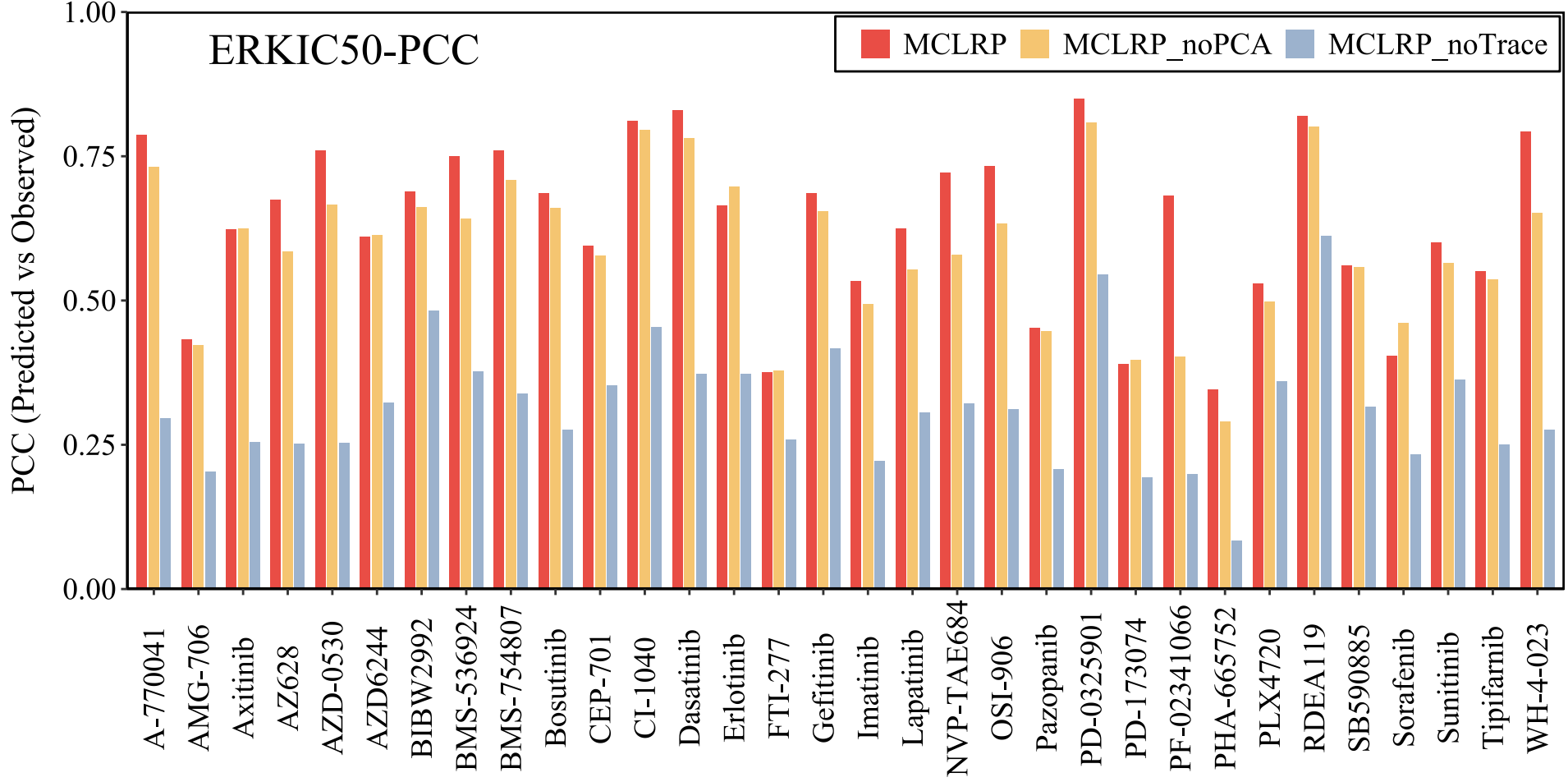

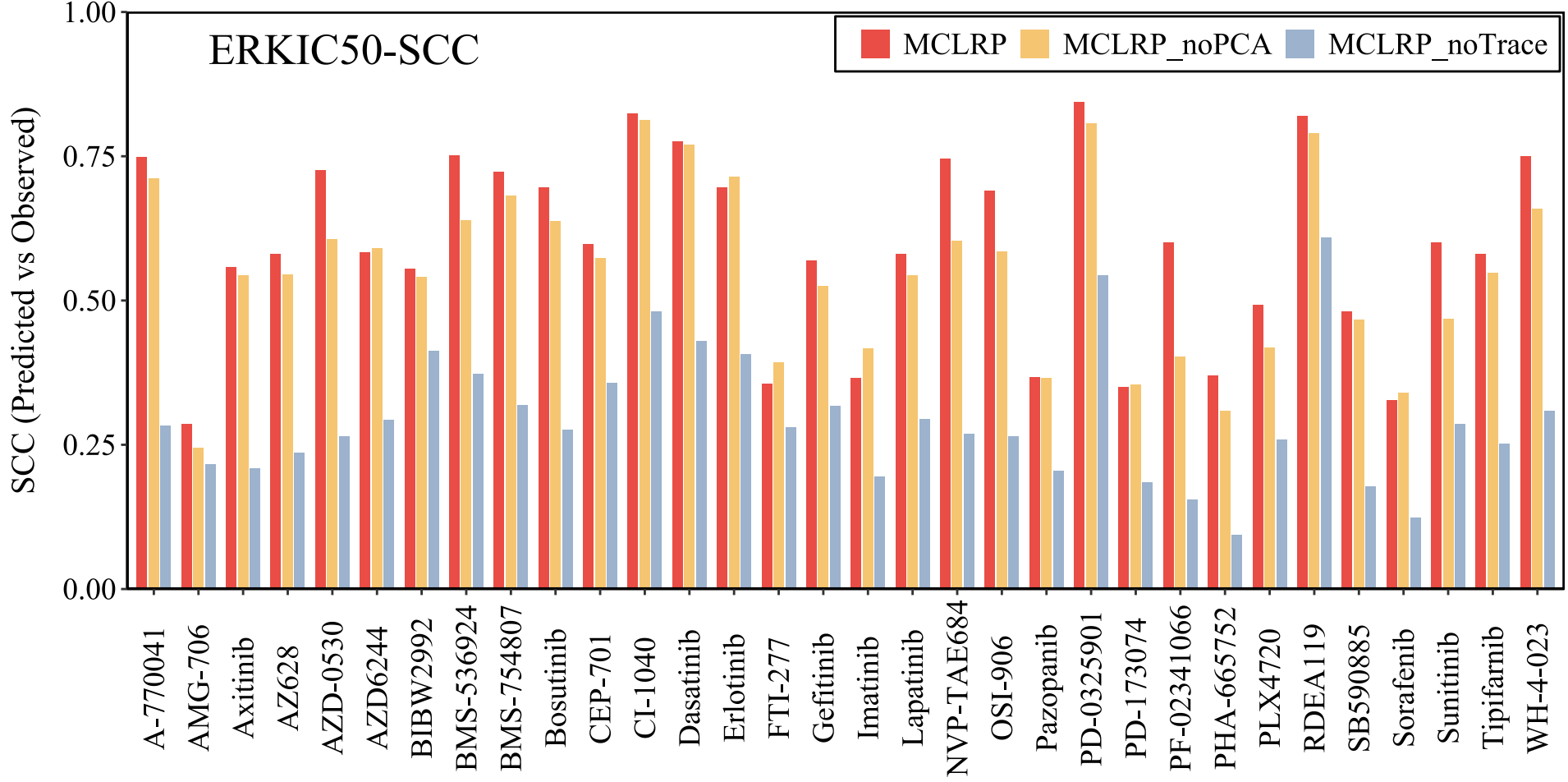

Supplement: Supplementary file 1 — Additional file 1. Figure S1. Prediction performance by the comparison of the five models in GDSC dataset, evaluated by PCC. Figure S2. Prediction performance by the comparison of the five models in GDSC dataset, evaluated by SCC. Figure S3. Prediction performance by the comparison of MCLRP, DeepIC50, and GeneVAE in the GDSC dataset. Figure S4. Comparative performance of MCLRP and its ablated variants on the GDSC dataset in terms of PCC and SCC. [file 12915_2025_2457_MOESM1_ESM.zip › Figure S4-Ablation Studies.pdf]
