# Supplementary material for: MCLRP: enhanced prediction of anticancer drug response through low-rank matrix completion and transcriptomic profiling
Source: BMC Biol. 2025 Dec 3;24:4. doi: 10.1186/s12915-025-02457-8 (PMC12781783; doi:10.1186/s12915-025-02457-8)
Supplement: Supplementary file 2 — Additional file 2. Tables S1-S4. The data for the predictive high sensitivity response values selected by different thresholds. Table S5. The data for the relationship between additional drugs and co-mutations on one or more genes. Tables S6-S9. The top 1000 genes corresponding to each drug in the five datasets. Table S10-S13. Functional enrichment analysis of the top 1000 genes for each drug and the terms with a Benjamin false discovery rate of < 0.05. Table S14. Conditions of use for the methods. [file 12915_2025_2457_MOESM2_ESM.zip › Table S14.docx]

Table 1. Conditions of use for the methods

| **Method** | **Algorithm applicability** | **Data characteristics** | **Data focus** |
| --- | --- | --- | --- |
| PCA | Unsupervised | Linear structure | Variance |
| LDA | Supervised | Linear structure | Type |
| FA | Unsupervised | Potential factors | Latent structure |
| ICA | Unsupervised | Mutual independence | Independence |
| t-SNE | Unsupervised | Nonlinear structure | Local similarity |
